# Modeling metabolic networks in *C. glutamicum*: a comparison of rate laws in combination with various parameter optimization strategies

**DOI:** 10.1186/1752-0509-3-5

**Published:** 2009-01-14

**Authors:** Andreas Dräger, Marcel Kronfeld, Michael J Ziller, Jochen Supper, Hannes Planatscher, Jørgen B Magnus, Marco Oldiges, Oliver Kohlbacher, Andreas Zell

**Affiliations:** 1 Center for Bioinformatics Tübingen (ZBIT), Wilhelm-Schickard-Institut für Informatik, Sand 1, 72076 Tübingen, Germany; 2 NNE Pharmaplan, Siemensstraße 21, 61352 Bad Homburg, Germany; 3 Forschungszentrum Jülich, Institut für Biotechnologie 2, 52425 Jülich, Germany

## Abstract

**Background:**

To understand the dynamic behavior of cellular systems, mathematical modeling is often necessary and comprises three steps: (1) experimental measurement of participating molecules, (2) assignment of rate laws to each reaction, and (3) parameter calibration with respect to the measurements. In each of these steps the modeler is confronted with a plethora of alternative approaches, e. g., the selection of approximative rate laws in step two as specific equations are often unknown, or the choice of an estimation procedure with its specific settings in step three. This overall process with its numerous choices and the mutual influence between them makes it hard to single out the best modeling approach for a given problem.

**Results:**

We investigate the modeling process using multiple kinetic equations together with various parameter optimization methods for a well-characterized example network, the biosynthesis of valine and leucine in *C. glutamicum*. For this purpose, we derive seven dynamic models based on generalized mass action, Michaelis-Menten and convenience kinetics as well as the stochastic Langevin equation. In addition, we introduce two modeling approaches for feedback inhibition to the mass action kinetics. The parameters of each model are estimated using eight optimization strategies. To determine the most promising modeling approaches together with the best optimization algorithms, we carry out a two-step benchmark: (1) coarse-grained comparison of the algorithms on all models and (2) fine-grained tuning of the best optimization algorithms and models. To analyze the space of the best parameters found for each model, we apply clustering, variance, and correlation analysis.

**Conclusion:**

A mixed model based on the convenience rate law and the Michaelis-Menten equation, in which all reactions are assumed to be reversible, is the most suitable deterministic modeling approach followed by a reversible generalized mass action kinetics model. A Langevin model is advisable to take stochastic effects into account. To estimate the model parameters, three algorithms are particularly useful: For first attempts the settings-free Tribes algorithm yields valuable results. Particle swarm optimization and differential evolution provide significantly better results with appropriate settings.

## Background

The metabolism of whole cells can be described as a network of metabolites and reactions interconverting these metabolites. To understand cellular systems, dynamic modeling of cellular processes has become an important task in systems biology [1-4]. Dynamic models describe the whole system and the state of each reacting species therein in a time-dependent manner [2]. Once such a model is constructed, several network properties can be derived, for instance stability, robustness, or the long-term behavior [5]. Furthermore, a well developed model provides a basis for predictions under different perturbations or varied environmental circumstances and can be applied to enhance the yield of desired metabolic products like certain amino acids [3].

To set up such models, appropriate rate laws have to be assigned to each reaction within the network. From these, a differential equation system that characterizes the rates of change of each reactant can be derived. However, setting up model equations is a difficult task. For many larger networks available in databases like KEGG[6,7] or METACYC[8] the reaction mechanism remains unknown. In many cases, reliable rate equations for the reactions are not known because these actually have to be derived for each catalyzing enzyme individually [9]. It is therefore a common approach to apply approximative rate laws, which characterize the most important features of the reaction rate. Many rate laws, which are either continuous or discrete, and either deterministic or stochastic [2], have been proposed for this purpose. Several examples of each group exist such as probabilistic [10,11], phenomenological [5,12-15], or semi-mechanistic approaches [5,16].

A second problem arises whenever a dynamic model of biochemical systems is created, because any such model contains a certain number of parameters like the reaction rates, Michaelis constants or the limiting rate as well as constants describing the influence of certain inhibitors [17-19] or, in stochastic systems, the reaction propensity [20]. Except for phenomenological models like power law approximations [21], linlog [12,22], or loglin kinetics [13,23], the parameter values can often be measured. However, this procedure is time-consuming, expensive, and often impractical. Online databases like the Brunswick Enzyme Database (BRENDA) [24-26] provide measured parameter values for many enzymes, but variations in the experimental settings, under which these and the time series measurements for the system under study were obtained, limit the applicability of these values for modeling purposes. In addition, it was observed that there are differences between parameters measured *in vivo *and *in vitro *[[27], p. 461]. The application of computational methods to optimize model parameters regarding the fit error has therefore become an important task in the model identification process [28-31]. In this connection, the optimizer tries to minimize the distance between measured values or values created *in silico *and the simulated time course for each reacting species by varying the model parameters. The smaller this distance is, the higher is the quality of a possible solution for one parameter set. This quality measure is often called the "fitness" of the parameter set. As an exhaustive search for the best solution is computationally not possible, heuristic optimization methods try to find the global optimum of the system. Usually, metabolic systems are analytically hard or infeasible to solve. Often those systems are non-convex or multimodal, i. e., contain numerous local optima, and the gradient cannot be computed easily. Biologically inspired optimization procedures like Evolutionary Algorithms (EAs) are known to handle even highly nonlinear optimization problems [32-34]. Many such optimization algorithms are freely available in several software packages [35-38] or included in commercial toolboxes [39].

During the last few decades, manifold derivatives of EAs have been proposed. Each of them has certain advantages and is therefore more or less appropriate for a special problem. Their development was driven by analogies to natural phenomena such as Darwinian evolution (genetic algorithm [40], evolution strategy [41], differential evolution [42]), hill climbing [43], the formation of crystal structures in metallurgy (simulated annealing [44]), or the swarm intelligence idea (particle swarm optimization [45,46]). Each one of these optimization procedures provides several settings that influence its performance; for instance, the temperature in simulated annealing, the crossover probability in genetic algorithms, or the population size in particle swarm optimization. For a detailled introduction to all heuristic optimization procedures used in this work [see Additional file 1].

In several studies, heuristic optimization procedures like EAs have been applied successfully to biochemical systems after these have been translated into sets of differential equations [32-34,47-50]. Most of the time standard settings for the optimization procedures are used. An analysis of these settings does, in fact, enable enhancement of the performance of the optimization process [33,47,48]. In many cases, a lack of time prevents researchers from systematically benchmarking these settings. In order to improve the model quality, however, this would be necessary. The resulting model systems, including the identified parameters, are often used to derive network properties like the long-term behavior or to perform steady-state analyses [49-53] of the system, but only in few cases detailed and specially derived rate laws could be applied to deduce a model system [49,50]. Many studies are available, in which a single type of approximative rate equation is applied to set up a model – in many cases without a comparison with alternative approaches. Guthke *et al*. modeled the amino acid metabolism of primary human liver cells using a phenomenological approach [51,53] whereas Liu and Wang used S-systems [21,54,55] for their biochemical models [56]. Magnus *et al*. applied linlog kinetics [12,22] to model the valine and leucine metabolism in *C. glutamicum *[52].

Very few studies compare alternative modeling approaches to investigate their applicability for the specific problem [9,34]. While Spieth *et al*. studied whether *in silico *time series data generated with certain model systems can be reproduced crosswise with other ones [34], Bulik *et al*. analyzed properties like stability when detailed kinetic equations within a system are replaced by approximative ones [9].

An investigation of both, alternative modeling approaches together with a systematical benchmark of the settings of optimization procedures, was rarely done. The disposability of software tools, which assign rate equations more or less automatically [57-61], requires the user to be especially aware of the properties of different modeling approaches and the possible quality that can be achieved with a certain type of kinetic models. Since automatically created models have already been published [62], and attempts have been made to scale up this modeling process to derive even genome-scale kinetic models automatically [63], these properties are of paramount importance. Summarizing, to construct a mathematical description of a biochemical reaction system the modeler has to consider at least two central questions:

1. Which rate laws are the most appropriate ones for the specific purpose?

2. Which optimization procedure performs best on the problem class of parameter inference?

Once a model has been created, the choice of initial conditions represents a further important question for an appropriate simulation of the model. Fundamentally, the system can be written as an initial value problem, sometimes referred to as a single-shoot approach [30], or as a multiple-shooting problem [31,64]. Here, a single-shoot strategy is employed using the steady-state concentrations of the participating metabolites as initial values.

**Figure 1:**
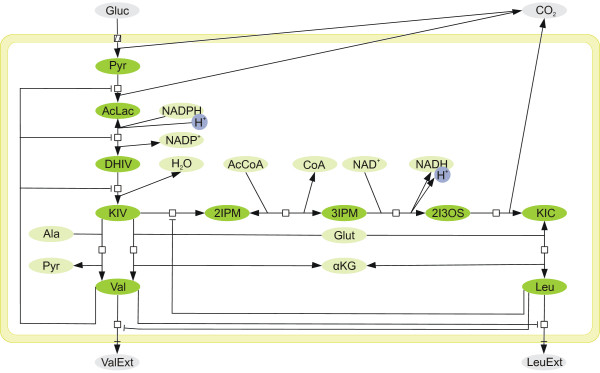
**Process diagram of the biosynthesis of valine and leucine in *C. glutamicum***. The pathway shown here is constructed using the information from both the KEGG and the METACYC databases. Metabolites outside the cell are not directly included in the model system. As 2-ketoisovalerate (KIV) is the starting point for both products, its production and degradation are highly regulated. For this purpose four feedback inhibitions control the reactions linked to this intermediate. Val and Leu can be transported out of the cell when not needed thereby competing for a free binding site in the transporting enzyme. Note that enzymes are omitted from this process diagram for the sake of a clear arrangement of the participating species.

This work addresses both questions and tries to identify an optimal model for a well-studied example network: the metabolism of L-valine (Val) and L-leucine (Leu) in *Corynebacterium glutamicum*, an aerobic gram-positive bacterium which is used to produce about two million tons of amino acids per year [65]. For this reaction network (see Figure 1 and Table 1) we construct seven alternative systems of differential equations based on four rate laws. These are the generalized mass action rate law [5], Michaelis-Menten equation, and convenience kinetics [16] as well as the stochastic Langevin equation [10,11]. To evaluate the influence of irreversible reactions, we construct two models for each deterministic rate law: one in which all reactions are considered reversible and a second one in which only two reactions are considered reversible. In a two-step benchmark we systematically examine eight optimization algorithms to estimate the parameters of all models. In a coarse-grained trial all algorithms are applied to all model systems with standard settings. In the fine-grained benchmark, alternative settings of the algorithms are evaluated to improve their optimization performance on the best models. We focus on nature-inspired heuristic optimization procedures [see Additional file 1], namely, Hill Climber (HC), Simulated Annealing (SA), Genetic Algorithm (GA), Evolution Strategy (ES), Differential Evolution (DE), Particle Swarm Optimization (PSO), and Tribes. A random (Monte Carlo) optimization serves as a general reference algorithm.

**Table 1:** The reaction system in more detail

No	Reaction	Enzyme	Inhibitor
*R*_1_	2 Pyr → AcLac + CO_2_	AHAS	Val
*R*_2_	AcLac + NADPH_2 _⇌ DHIV + NADP^+^	AHAIR	Val
*R*_3_	DHIV → KIV + H_2_O	DHAD	Val
*R*_4_	KIV + Glut → Val + *α*KG	BCAAT_ValB_	
*R*_5_	KIV + Ala → Val + Pyr	BCAAT_ValC_	
*R*_6_	Val → Val_ext_	Trans_Val_	Leu
*R*_7_	KIV + AcCoA → IPM + CoA	IPMS	Leu
*R*_8_	IPM + NAD^+ ^→ KIC + NADH_2 _+ CO_2_	IPMDH	
*R*_9_	KIC + Glut ⇌ Leu + *α *KG	BCAAT_LeuB_	
*R*_10_	Leu → Leu_ext_	Trans_Leu_	Val

The reactions in KEGG are lumped together resulting in this reaction scheme which is in accordance with METACYC except for the question of irreversibility, and apart from *R*_2 _and *R*_9_, which are reversible.

## Results and discussion

### Mathematical models

Figure 1 shows the biosynthesis of Val and Leu in *C. glutamicum*, starting with pyruvate (Pyr). The reactions of this pathway are summarized in Table 1. In *R*_3_, one substrate (DHIV) turns into one product (KIV) when neglecting the influence of the second product, water, which is plentiful in the cell. Therefore, we can apply a Michaelis-Menten equation to model the kinetics of this reaction. The transport of Val and Leu through the cell wall (*R*_6 _and *R*_10_) has exactly one substrate and one product and can, therefore, also be modeled using the Michaelis-Menten approach. All other reactions in the network cannot be modeled using this classical approach because multiple substrate or product molecules are involved. Therefore, approximative rate laws can be applied to these reactions. Here we employ one stochastic and three deterministic rate equations. Approximative rate laws can be used for the three reactions with a Michaelis-Menten mechanism as well.

The reversibility of the reactions constitutes another important question to be solved before modeling. As transport through the cell wall removes both products from the cellular system, we assume that there is no reverse reaction (an uptake of Val or Leu) and, hence, consider both reactions irreversible. The two reactions *R*_2 _and *R*_9 _are reversible [6-8,52]. We let the optimization procedure "decide" if the remaining reactions should be modeled in a reversible or irreversible way. To this end, we construct one "reversible" and one "irreversible" alternative model for each rate law, keeping only the two known reactions *R*_2 _and *R*_9 _reversible.

In this way we derive the following seven models based on four approaches for the reaction velocity on this pathway. Details and the formulas can be found in Methods and [see Additional file 2].

**GMAKr **Pure generalized mass action kinetics, in which all reactions apart from *R*_6 _and *R*_10 _are modeled reversibly, with 24 parameters.

**GMAKi **Pure generalized mass action kinetics, in which only the two reactions *R*_2 _and *R*_9 _are considered reversible, with 18 parameters.

**GMMr **Like GMAKr but with three Michaelis-Menten equations for reactions *R*_3_, *R*_6 _and *R*_10_. This model contains 31 parameters. In the GMAKr model the influences of all enzymes are neglected and hidden in the rate constants, which is an oversimplification of the biochemical process. The model comes closer to the biochemical process when inserting Michaelis-Menten equations for the three reactions *R*_3_, *R*_6_, and *R*_10_.

**GMMi **Like GMMr but with only two reversible reactions *R*_2 _and *R*_9_, leading to 24 parameters.

**CKMMr **Convenience kinetics with three Michaelis-Menten equations as in GMMr. All reactions apart from *R*_6 _and *R*_10 _are considered reversible leading to 59 parameters. The convenience rate law is also an approximation of the biochemical process. Therefore, we do not construct a pure convenience kinetics model of the whole system but apply Michaelis-Menten kinetics whenever this is possible (*R*_3_, *R*_6 _and *R*_10_).

**CKMMi **Convenience kinetics with three Michaelis-Menten equations as in model GMMi, in which only the two reactions *R*_2 _and *R*_9 _are considered reversible, with 41 parameters.

**LANG **To demonstrate the possibility of large-scale parameter optimization, even for stochastic models, and to model the effects of random fluctuations in the metabolite concentrations, we consider a stochastic description as well. Based on the Langevin equation, this system contains 24 parameters.

In a glucose stimulus-response experiment 47 measurements are taken for 13 metabolites on this pathway (for details see Methods). The parameters of all models are calibrated with regard to these data.

### Fine-tuned optimization algorithms and models

**Figure 2:**
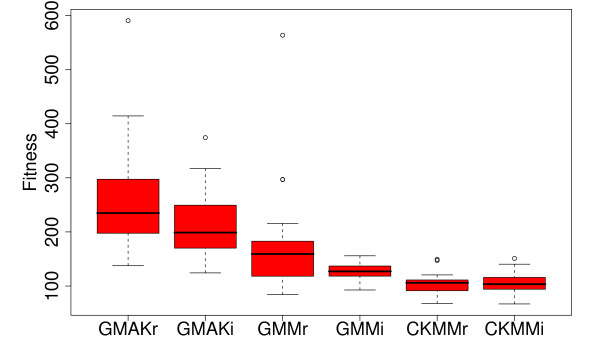
**Parameter identification using random optimization**. The Monte Carlo method constitutes the simplest way to optimize the parameters within a model. Thereby this method "dices" a random solution within the search space and logs the best solution found. This procedure is repeated twenty times in fifty multi-starts using 100,000 evaluations. The more complex the model the better is the solution found by the Monte Carlo method (RSE 66.920 for the CKMMi model), but still this method cannot approach the quality of independently generated splines (RSE 19.670).

**Figure 3:**
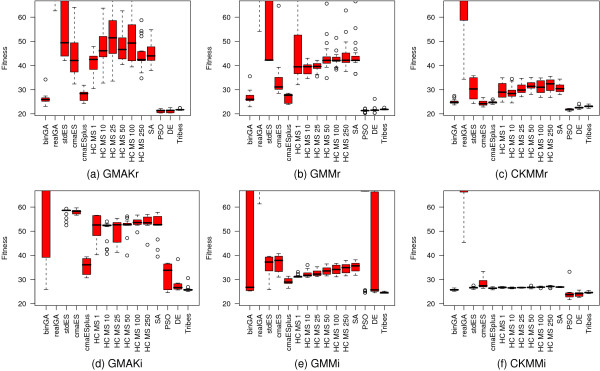
**Comparison of the standard optimization algorithms applied to each deterministic model**. Five algorithms show outstanding results on the reversible models. These are binGA, cmaESplus, DE, PSO, and Tribes. On the irreversible models, binGA does not yield such good average results, but is still capable of finding good local optima. For these models, cmaESplus and Tribes also find pre-eminent results, whereas PSO and DE are among the best algorithms, except for the GMM models. The real-valued GA performs worst in all cases. The hill climbers show differences in their effectiveness when invoked with various numbers of multi-starts but cannot compete with the other algorithms. The term "fitness" is used to define a quality measure of possible solutions, which is minimized.

In the next step the parameters of all seven mathematical models have to be estimated. In this process the relative distance between simulated model data and experimental data serves as a quality measure (fitness) of an estimated parameter set. Note that the error between the measurements and the model simulation is to be minimized, so the quality of the solutions increases with decreasing error values. A random optimization (Monte Carlo search) of the models (Figure 2) yields relative differences between measurements and model systems that are at least three times higher than the difference between the measurements and uncoupled cubic approximation splines. Therefore, we apply the nature-inspired heuristic optimization procedures HC, SA, GA, ES, DE, PSO, and Tribes to all seven models with standard settings (Table 2; details in Methods section). Apart from only some minor exceptions for the two irreversible models, five algorithms turn out to be especially useful (Figure 3 and Table 3). These are the binary-valued Genetic Algorithm (binGA), Evolution Strategy with covariance matrix adaptation [66] (cmaES) with elitism (plus strategy), PSO, DE, and Tribes. The performance of the lattermost procedure cannot be further improved as this algorithm is a settings-free derivative of PSO. Hence, we study the influences of various settings on the capabilities of the other four procedures aiming to improve the fit of the model to the data for each deterministic model.

**Table 2:** Settings for the standard algorithms in detail

Algorithm	Population	Mutation	Crossover	Selection
Monte Carlo	50	no	no	Best

Hill Climber	1, 10, 25, 50, 100, 250	Fixed Step *σ *= 0.2, *p*_*m *_= 1	no	Best

binGA	250	one-point, *p*_*m *_= 0.1	one-point, *p*_*c *_= 0.7	Tournament, group of 8

realGA	250	global, *p*_*m *_= 0.1	UNDX, *p*_*c *_= 0.8	Tournament, group of 8

stdES	(5, 25)	global, *p*_*m *_= 0.8	discrete one-point, *p*_*c *_= 0.2	Best

cmaES	(5,^+^25)	CMA, *p*_*m *_= 1	no	Best

SA	250	linear annealing schedule, *α *= 0.1, initial *T *= 5		Best

DE	100	current-to-best/1, *λ *= *F *= 0.8, *CR *= 0.5		

PSO	100	star topology, *ϕ*_1 _= *ϕ*_2 _= 2.05, *χ *= 0.73		

This overview lists the standard settings for the optimization procedures used to infer the parameters in the differential equation systems. The algorithms DE and PSO do not follow the general scheme of mutation and crossover. The Tribes algorithm is not listed here as it was designed to be a settings-free derivative of PSO. The cmaES is used with and without elitism (plus or comma strategy). For details, see Methods and [see Additional file 1].

**Table 3:** Preliminary test results

GMAK, reversible					GMAK, irreversible				
	
Min.	Algorithm	Average	Std. Dev.	Algorithm	Min.	Algorithm	Average	Std. Dev.	Algorithm
			
20.334	PSO	21.190	0.576	PSO	24.587	PSO	25.967	1.171	Tribes
20.335	DE	21.228	0.756	DE	25.006	Tribes	29.502	9.610	DE
21.401	Tribes	21.725	0.275	Tribes	25.683	DE	33.169	10.143	PSO
23.097	binGA	26.106	2.204	binGA	25.981	binGA	35.670	3.125	cmaESplus
24.321	cmaESplus	27.598	2.091	cmaESplus	30.704	cmaESplus	50.663	4.138	HC MS 10
GMM, reversible					GMM, irreversible				
	
Min.	Algorithm	Average	Std. Dev.	Algorithm	Min.	Algorithm	Average	Std. Dev.	Algorithm
			
20.312	PSO	21.272	0.461	PSO	24.477	Tribes	24.654	0.282	Tribes
20.407	DE	21.711	1.153	DE	24.499	DE	37.696	19.374	DE
21.590	Tribes	21.887	0.243	Tribes	24.553	PSO	41.529	21.768	binGA
22.913	binGA	26.742	2.711	binGA	25.266	binGA	31.136	9.052	cmaESplus
23.890	cmaESplus	26.624	1.851	cmaESplus	25.812	stdES	31.338	0.546	HC MS 1

CKMM, reversible					CKMM, irreversible				
	
Min.	Algorithm	Average	Std. Dev.	Algorithm	Min.	Algorithm	Average	Std. Dev.	Algorithm
			
20.882	PSO	21.773	0.352	PSO	21.632	PSO	23.968	0.931	DE
21.821	DE	22.633	0.562	DE	22.651	DE	24.624	0.315	Tribes
22.258	Tribes	23.079	0.464	Tribes	24.191	Tribes	25.761	0.331	binGA
22.829	cmaES	24.341	1.026	cmaES	25.152	binGA	26.434	0.339	cmaESplus
23.687	binGA	24.736	0.557	cmaESplus	25.738	cmaESplus	26.539	0.210	HC MS 10

For all deterministic models (reversible models on the left, irreversible models on the right) the five absolute best algorithms and the five average best algorithms with standard deviations are listed.

Due to the generally better performance of the three reversible models, we examine alternative settings for the optimization procedures only for these models and subsequently apply the best setting found to each alternative irreversible model. The most promising settings are used to optimize the stochastic model as well.

**Figure 4:**
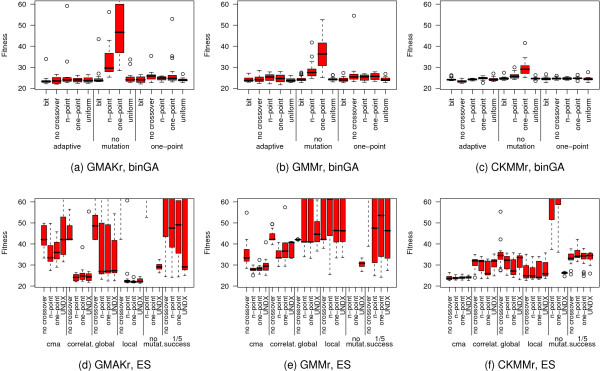
**Benchmark of various combinations of mutation and crossover operators**. The six box plots depict the dependency of fitness on a multitude of combinations of mutation and crossover operators available for binGA (a-c) and ES (d-f). All plots are limited to a fitness of 60. The more detailed the model the better the overall fitness that can be obtained. For binGA almost all combinations of mutation and crossover operators yield good fitness values. The only two exceptions are no mutation with one- or *n*-point crossover, which shows the worst fitness on all three models. The ES is more sensitive to alternative combinations of mutation and crossover operators. The abbreviations cma, correlat., no mutat., and 1/5 success stand for covariance matrix adaptation, correlated, and no mutation and for the 1/5^th ^success rule. The elaborated comparison shows that some settings of the ES lead to equally good or even better results than that of binGA. However, most ES-settings cannot compete with the fitness values found for most binGA settings.

**Figure 5:**
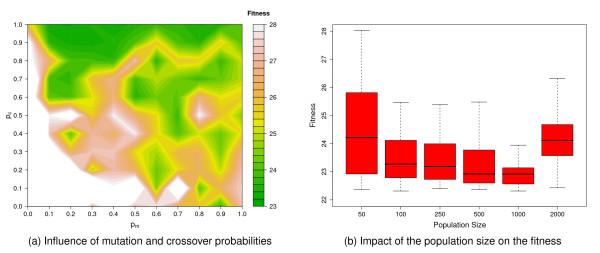
**Optimizing the settings for binGA on the reversible GMAK model**. The best combination of mutation and crossover operators for binGA on the reversible GMAK model is adaptive mutation and bit-simulated crossover. For this pair the influence of the mutation and crossover probabilities *p*_*m *_and *p*_*c *_is examined and plotted in (a). Each experiment is repeated 20 times. The combination *p*_*m *_= 0.2 and *p*_*c *_= 1.0 improved the average fitness most successfully. Figure 5(b) depicts the impact of the population size for this setting. The variance decreases with an increasing population size and reaches its minimum at 1,000. However, the best single result cannot be surpassed.

Taking a closer look at the effects of alternative mutation and crossover operators on binGA and ES (Figure 4) reveals that the more detailed CKMMr model can be fitted to the data with almost any combination of both operators, whereas the other two reversible models show larger differences. BinGA especially provides good performance with almost every operator combination. The only two exceptions are no mutation combined with one- or *n*-point crossover. The influence of these operators on ES is much stronger and more problem-specific. Some settings improve the performance of ES, but most result in significantly worse fitness values. In contrast to binGA, for which the combination of adaptive mutation with one-point or bit-wise crossover or adaptive mutation without crossover provides an improved average performance, the plots in Figure 4(d) through Figure 4(f) do not show such a general trend for ES. Thus, we evaluate the influence of the mutation and crossover probabilities *p*_*m *_and *p*_*c *_on only binGA to identify the best ratio. The plot of the resulting fitness landscape for the GMAKr model was limited to a fitness of 28 (Figure 5(a)). Hence, areas with a worse performance are shown in white. The best combination *p*_*m *_= 0.2 with *p*_*c *_= 1.0 is the starting point for investigating the influence of different population sizes. We vary the population sizes from 50 to 2,000 (Figure 5(b)). The larger the population, the smaller is the variance within this population. However, if the population is too large, this variance increases again. Although a larger population leads to smaller variances, statistically, it does not help to find a better total solution for the optimization problem.

**Figure 6:**
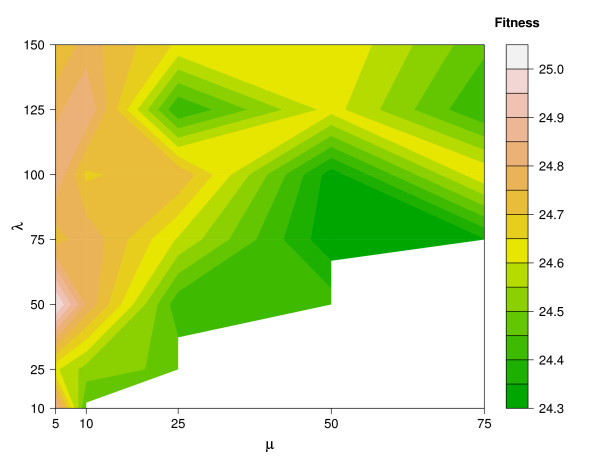
**Influence of the population size on the performance of cmaESplus for the reversible CKMM model**. The Evolution Strategy with covariance matrix adaption and elitism is one of the most promising optimization strategies among the standard algorithms. Here, we examine whether its capabilities can be improved by another choice of the parameters (*μ *+ *λ*). All experiments are repeated 20 times. The fitness landscape shows a minimum in the average values at (50 + 75).

**Figure 7:**
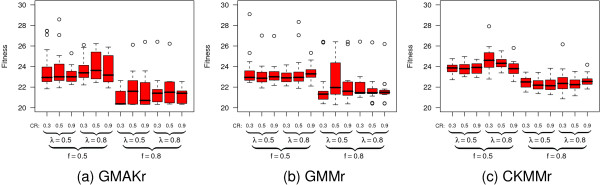
**Comparison of a multitude of settings for differential evolution on the three reversible models**. The settings of DE are studied on the reversible models. All plots suggest that the choice *F *= 0.8 is more appropriate than *F *= 0.5, whereas *CR *and *λ *influence the results less clearly.

**Figure 8:**
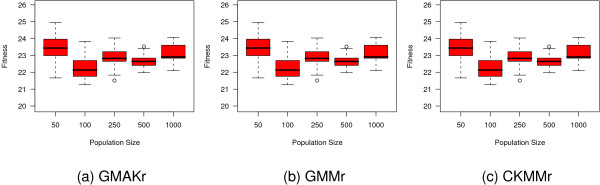
**Influence of various population sizes on the performance of differential evolution**. The settings *F *= 0.8, *λ *= 0.5, and *CR *= 0.3 are found to be most suitable for the GMAKr and GMMr model, whereas *CR *= 0.9 performs slightly better on the CKMMr model. Using these settings, the influence on the population size is studied. The boxplots suggest that a population size of 100 yields the best median results over 20 repeats.

Due to the fact that cmaESplus leads to reasonable results on each model and also that there is no general trend for alternative combinations of other mutation and crossover operators, we pick the CKMMr model to examine the influence of population size (*μ *+ *λ*). The value of *μ *represents the number of parents in the population from which, in each generation, *λ *offsprings are created. Figure 6 depicts the resulting fitness landscape. Combinations of *μ *and *λ *with *μ *> *λ *are left out and occur as a white area within the landscape. Larger population sizes lead to better average fitness values. ES achieves its best average and total performance for (25 + 125). The combination (50 + 75) leads to only a slightly worse average and total fitness. These two settings clearly outperform all other combinations. By varying the values for *F*, *λ*, and *CR *we test how to improve the performance of DE on each reversible model. Generally, the choice of *F *= 0.8 leads to a better performance than *F *= 0.5 (Figure 7). The influence of the remaining two parameters is less clear. Hence, we pick the best setting for each model and evaluate the influence of different population sizes (Figure 8). A population with 100 individuals performs best on each model according to the median.

**Figure 9:**
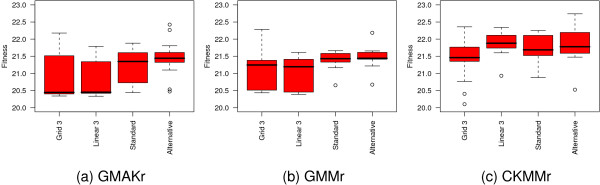
**Influence of the settings on particle swarm optimization**. Besides the standard star topology, a grid 3 and a linear 3 topology is tested on the three reversible models with *ϕ*_1 _= *ϕ*_2 _= 2.05. Furthermore, the alternative setting *ϕ*_1 _= 2.8 and *ϕ*_2 _= 1.3 is applied to the star topology. The grid topology performs best according to the median for the GMAKr and the CKMMr models, but is slightly worse than the linear topology on the GMMr model. All experiments are repeated 20 times.

**Figure 10:**
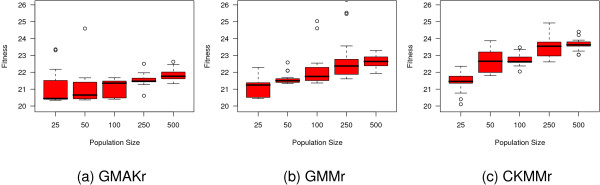
**Influence of the population size on the performance of PSO with grid 3 topology**. An increasing population size cannot improve the performance of PSO.

To hone the performance of PSO we alter both strategy parameters *ϕ*_1 _and *ϕ*_2 _on the star topology and apply a grid 3 and linear 3 topology using the standard values for *ϕ*_1 _and *ϕ*_2 _(Figure 9). On the three reversible models the grid 3 topology performs slightly better than all other settings. Hence, we test how an alternative population size influences its capacity. Figure 10 depicts the results of this experiment where we vary the size of the population in the intervall from 25 to 500. A larger size lowers the quality and a small population of 25 individuals is confirmed to be the best choice.

### Comparison of the performance of the optimization algorithms

An overview of the most successful optimization algorithms together with their best suited settings can be found in Table 4. Tribes is not the very best optimization algorithm but yields meaningful results for all models. As a settings-free procedure, Tribes is a good choice if there is no time to examine alternative adjustments. The standard PSO algorithm yields the best median fitness for the CKMMr model with 21.687. On the GMAKr model, DE with *F *= 0.8, *λ *= 0.5, and *CR *= 0.3, and a population size of 100 gives the best median fitness of 20.369 for this model. This is almost 0.9 better than standard PSO. DE yields a median fitness of 22.196 on the CKMMr model when set to *F *= 0.8, *λ *= 0.5, *CR *= 0.5, and a population size of 100. Both algorithms also perform well on the GMMr and the Langevin model (in total, average, and median). Hence, they are an advisable choice when optimizing the parameters of various mathematical models of biological systems.

**Table 4:** Statistics on the most successful runs of each main optimizer

Model	reversible			irreversible			Algorithm	Population
				
	Min	Avg	Std. Dev.	Min	Avg	Std. Dev.		
GMAK	20.326	20.742	0.501	25.745	34.472	15.285	PSO linear 3, *ϕ*_1 _= *ϕ*_2 _= 2.05	25
	20.403	21.787	1.297	25.183	31.694	13.255	DE, *f *= 0.8, *λ *= 0.5, *CR *= 0.3	100
	21.975	23.812	1.604	24.741	49.045	21.285	binGA, adaptive MUT, no CROSS	250
	24.321	27.598	2.091	30.704	35.670	3.125	cmaES	(5+25)

GMM	20.280	22.818	2.186	24.857	27.978	9.146	DE, *f *= 0.8, *λ *= 0.5, *CR *= 0.5	100
	20.312	21.272	0.461	24.553	58.957	17.253	PSO star, *ϕ*_1 _= *ϕ*_2 _= 2.05	25
	21.649	24.628	1.801	24.616	40.896	25.881	binGA, adaptive MUT, one-point CROSS	250
	23.890	26.624	1.851	26.414	31.136	9.052	cmaES	(5+25)

CKMM	20.100	21.434	0.563	21.511	26.077	7.729	PSO grid3, *ϕ*_1 _= *ϕ*_2 _= 2.05	25
	20.862	22.499	1.119	21.763	23.603	1.268	DE, *f *= *λ *= 0.8, *CR *= 0.3	100
	21.431	23.222	1.066	25.632	28.055	2.697	cmaES	(10, 50)
	22.092	23.353	0.666	25.040	25.346	0.176	binGA, adaptive MUT, no CROS	100

For each model the minimal fitness and the corresponding standard algorithm are listed. The algorithm that achives the best average fitness and the corresponding average fitness are written in the last two columns together with the standard deviation. On the Langevin model, PSO with star topology, *ϕ*_1 _= *ϕ*_2 _= 2.05 and a population size of 100 is the most successfull algorithm with a fitness of 20.716.

### Comparison of the modeling approaches

**Figure 11:**
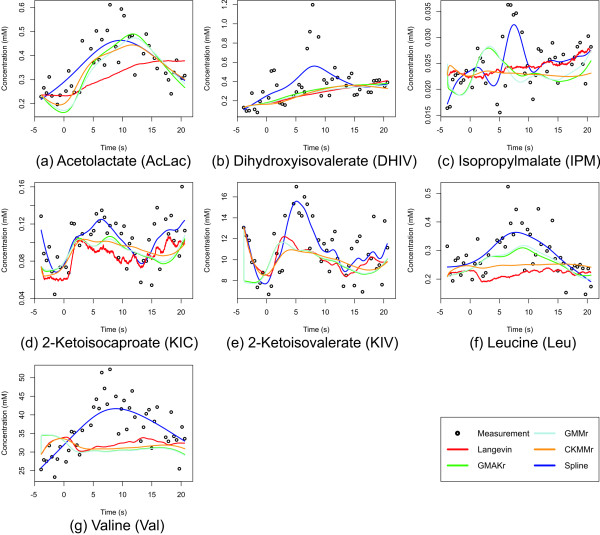
**The best fit of all reversible deterministic and the Langevin models**. Shown are the Langevin model, the measurements, and all reversible deterministic models. For a better visualization, splines are added to the data as well to help imagin how a perfect model curve would look. However, as can be seen, biological measurement data always shows fluctuations. The splines shown here are uncoupled, one individual spline for each metabolite. Thus, there is no underlying biological model motivating these curves. The irreversible models mostly result in straight lines through the data and are hence biologically implausible. For the sake of a clearer recognition of the reversible models, we omit these curves from the plot, [see Additional file 2] for the irreversible models.

Figure 11 depicts the measurement data together with the best simulation results of each reversible deterministic and the Langevin model.

To evaluate which model is the most promising one we consider the following three criteria:

1. Fit of the model to the data

2. Number of model parameters

3. Computational time for simulation (computational complexity, weak criterion)

Each reversible deterministic model can be fitted to the data with a similar deviation from the measurements. The irreversible alternatives show a significantly higher deviation. Only the irreversible CKMM model is able to fit the data almost as well as the reversible models. However, most curves resulting from all of the irreversible models tend to become straight lines through the measurements, and thus behave in a biologically implausible manner [see Additional file 2]. This suggests that the irreversible models are unable to follow the dynamics of the system due to their fewer degrees of freedom. The rather abstract reversible models are able to tackle possible side effects of reactions not included in this reaction system and simulate them in terms of the reverse reaction. These models also consider the fact that, in biological systems, reaction products are normally not completely absent. Their concentration may be low, but they still take part in the reaction, in some cases giving a kind of feedback to the reactants [[19], pp. 312–313]. Therefore, the irreversible models are generally not competitive with respect to the data fit. From the three remaining reversible models, the CKMMr model achieves the best fit to the data, with 20.100, followed by GMMr model at 20.280 (worse by 0.180), and the GMAKr model at 20.326 (slightly worse by a further 0.046). The difference in the best model fit between the CKMMr and the GMAKr models is only 0.303. Hence, all three reversible models can be fitted to the data with a similarly small relative squared error (RSE, see Methods). A comparison of the best model fit 20.100 (CKMMr) to the fitness of the independently computed splines (19.670) evinces a difference of only 0.430. When considering the number of parameters (criterion 2), the GMAKr model shows a clear advantage with its 24 parameters compared to the 31 of the GMMr model or even 59 of the CKMMr model.

When choosing the parameter values for the GMAKr model completely randomly, this system can hardly be integrated without step size adaptation. Therefore, it is necessary to identify meaningful ranges of kinetic parameters within BRENDA [24-26]. A low-value initialization is necessary to assure numerically stable initial populations for the optimization procedures. For the other models this happens only if the parameters are chosen from implausibly large ranges.

The last criterion, the computation time, also depends on the complexity of the model but not necessarily on the number of parameters. The GMAKr model requires the smallest number of mathematical operations, followed by the GMMr model. The most complicated model is the CKMMr model. The average evaluation time over 100,000 repeats with randomly chosen parameters is 49 ms for the GMAKr model, 101 ms for the GMMr model and 151 ms for the CKMMr model. For hardware details see the Hardware Configuration section.

In order to take the effects of random fluctuations into account, one has to use stochastic models of the chemical reaction system. However, the most general approach, the chemical master equation [67], can hardly be solved numerically for larger systems [20]. Taking the number of parameters (criterion 2) and the computational costs (criterion 3) into account, the Langevin model is the most suitable formalism to consider the effects of random fluctuations while still providing an acceptable performance. Since the model can be stated in a way that allows it to be integrated with standard solvers for ordinary differential equations, the computational costs are of the same order of magnitude as the solution of the GMAKr model. However, care must be taken with respect to justification of the underlying simplifying assumptions [10]. The stochastic model equations of the biological system under consideration show no qualitatively different behavior in comparison to the deterministic model. The main reasons for this observation are found in the rather large molecule populations and the absence of points of instability in the allowed phase-space region.

### Parameter space analysis

Once a model has been optimized several times and locally optimal parameter sets for the model are available, an analysis of the space of potential solutions becomes possible. This allows deducing characteristics of the solution space aiming to reduce the model complexity and enhance the optimization performance. If, for instance, two parameters show a linear dependency or correlation to each other, one of these can be removed from the model. Another interesting experiment would be to determine new ranges for each parameter. This can be done if a certain parameter varies only in a very small range compared to its maximal possible range.

For each of the three models, GMAKr, GMMr, and CKMMr, we gather all parameter values from all optimization runs that lead to a fitness less than 25. In this way, we obtain one parameter matrix for each model, in which each column corresponds to one optimization run and each row stands for one parameter. We conduct three analyses on the best parameters on each reversible model (see Methods):

1. *Clustering *to identify groups of similar ranges or almost constant values and to visualize the values of each parameter.

2. *Variance analysis *to visualize the scattering of each parameter.

3. *Multiple correlation analysis *aiming at finding highly correlated parameters, some of which can be eliminated.

For Histograms showing the parameter distribution of the GMAKr, GMMr, and CKMMr models [see Additional file 2].

**Figure 12:**
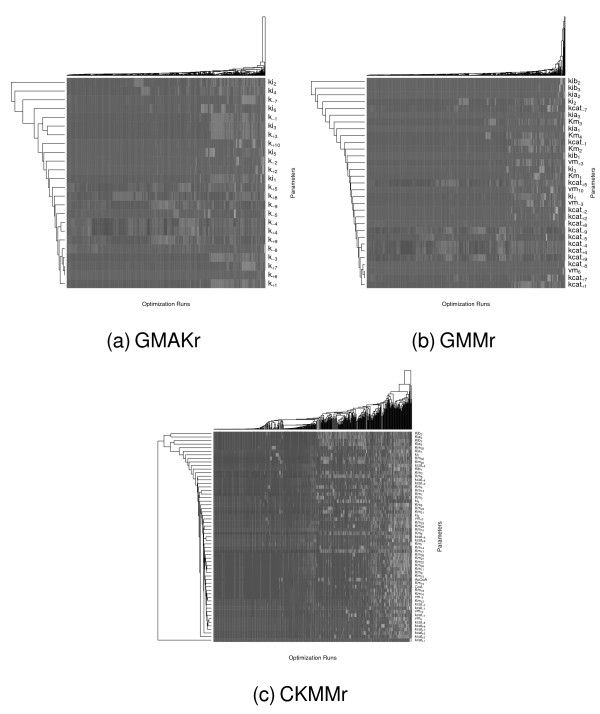
**Clustering of the best parameter values of all reversible models**. A cluster analysis is carried out on all parameter values that have a fitness of less than 25. Each row of the above heatmaps corresponds to one parameter of the respective model and each column gives one parameter set that has been obtained in one of the optimization runs. The cluster algorithm swaps rows and columns to group similar parameters and parameter sets next to each other. This procedure leads to stripes from the left to the right, meaning that many parameters are often set to similar values in all optimization runs. If there were rectangular blocks within the heatmaps, this would mean that some parameters are correlated, thus showing a similar behavior, but because this is not the case, all parameters are distinct from each other, which can also be seen from the flat dendrograms at the side of each heatmap. Also, the dark-colored figures show that most parameters are set to low values because zero corresponds to black. The lighter the color the higher the value. For details, histograms showing the parameter distribution for the three models [see Additional file 2].

*Clustering *groups similar parameters and similar optimization runs together. First, all parameters are reordered so that within the parameter vector all parameters with similar values over all optimization runs are placed next to each other. In the second step, all parameter vectors from every optimization run are swapped so that similar parameter vectors are located next to each other. Figure 12 graphically displays the results of the clustering approach (1). The heatmaps show all parameters on the *y*-axis and all optimization runs on the *x*-axis. The lighter the color, the lighter the value e of the parameter in the respective optimization run, with black representing values close to zero. As can be seen, there is a very flat hierarchy so there are no groups of parameters showing a similarity, but there are many parameters which are similar to their neighbor. A similar flat hierarchy can also be seen for the optimization runs. There is almost no relationship between the values of the parameters with respect to the experiments. This means that each parameter was optimized independently by the analyzed procedures. Some parameters show stripes within the heatmap of Figure 12. Stripes like these mean that the corresponding parameter barely varies in its value over all experiments. Note that these more or less constant parameters do not occur within the same cluster. All parameters of this type can either be replaced by their median thus reducing the complexity of the system, or the ranges of these parameters can be set to more restrictive values. However, the experiments are broken into two groups in all three models: The first group shows homogeneously distributed parameter values whereas the second one contains more differences. The level of differences in the second group rises with the complexity of the model.

**Figure 13:**
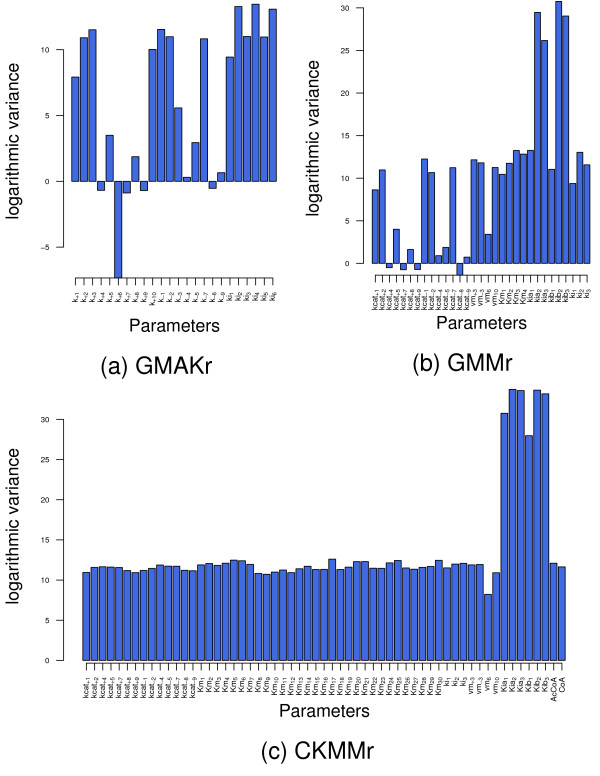
**Variance analysis of the best parameter values of all reversible models**. Each bar plot shows the variances of every parameter among the best optimization runs for the three reversible deterministic models. For the sake of a better visualization these are plotted with a logarithmic scale.

These results are confirmed by the *variance analysis *(2), whose results are shown in Figure 13. As can be seen, the higher the dimension of the optimization problem, i. e., the more complex the model is, the higher are the variances among the parameter set. This indicates that all parameters in the CKMMr model are allowed to vary within a rather large range. Such behavior is often referred to as a multimodal optimization problem. In contrast, the less complex models, GMAKr and GMMr, showed several dimensions of almost no variance. This corresponds to the observation made from the heatmaps that certain parameter values can vary only within a small range of values or even stay constant over multiple optimizations. Thus, the probability of finding multiple local optima increases with the model complexity. Particularly, all parameters which represent the impact of inhibitors exhibit noticeably large variances. The biological interpretation of this is that variations in strength and concrete mechanism of inhibition in one reaction can be balanced in terms of other reactions because this pathway contains four feedback inhibition mechanisms for this purpose (structural robustness).

**Figure 14:**
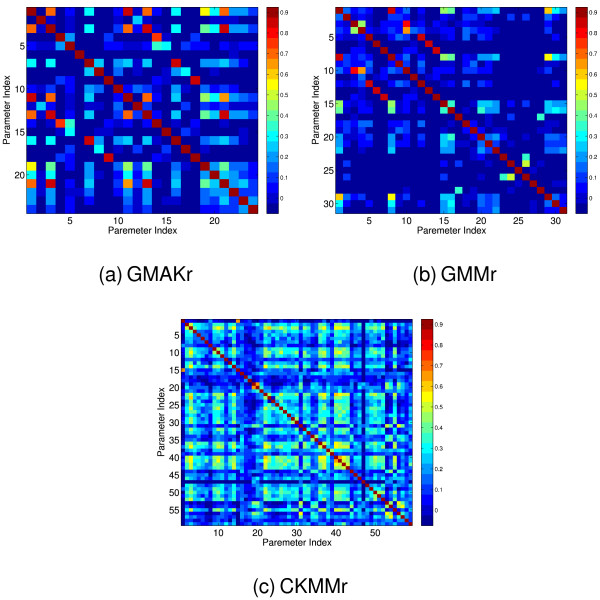
**The correlation among the parameters in all reversible models.** Each heat map shows to which extent each parameter is correlated with all other ones. The main diagonal shows the self correlation, which equals one. The correlation was computed using a multiple correlation analysis based on the Pearson correlation coefficient. If two parameters are highly correlated, one of both can be replaced by the other one hence reducing the system's dimensionality.

In order to identify linear dependencies between model parameters, we perform a *multiple correlation analysis *(3). For maximum generality, each parameter is assumed to possibly correlate with all other parameters of the system. Several highly correlated parameters are found in each model system. The correlation results are shown in Figure 14. All highly correlated parameters found exhibited significant variances as can be seen in Table 5 and Figure 13. We select a subset of parameters to be replaced by a linear regression model of highly correlated parameters. In this way the number of parameters is reduced by seven in the GMAKr model, by five in the GMMr model and by four in the CKMMr model. Subsequently, each model is optimized with the reduced parameter set, using the linear regression model for the non-optimized parameters. For this optimization, PSO is used because it is the the best-performing procedure in our benchmark. The results are shown in Table 5. The parameter reduction induced only a small loss of performance in each model, indicating that the original number of parameters does not reflect the true degrees of freedom of the system.

**Table 5:** Reduced reaction system and optimization results

Model	Replaced Parameter	Linear Regression Model	Mean Fitness ± Std. Dev.
GMAKr	*k*_+1_	*a*_1 _· $K_{3}^{\text{I}}$	21.4782 ± 0.3254
	*k*_+3_	*a*_2 _· $K_{3}^{\text{I}}$	
	*k*_-1_	*a*_3 _· $K_{3}^{\text{I}}$	
	*k*_-3_	*a*_4 _· $K_{3}^{\text{I}}$	
	*k*_+4_	*a*_5 _· *k*_-4_	
	*k*_+7_	*a*_6 _· *k*_-7_	
	*k*_+9_	*a*_7 _· *K*_-9_	

GMMr	*k*_-4_	*b*_1 _· *k*_+4 _+ *b*_2 _· *k*_+5_	21.9538 ± 0.0832
	*k*_+1_	*b*_3 _· *k*_-1 _+ *b*_4 _· $K_{1}^{\text{I}}$	
	*k*_+7_	*b*_5 _· *k*_-7_	
	*k*_+8_	*b*_6 _· *k*_-8_	
	*k*_+9_	*b*_7 _· *k*_-9_	

CKMMr	$k_{+1}^{\text{cat}}$	*c*_1 _· $K_{{\left[\text{Pyr}\right]}_{1}}^{\text{M}}$	22.7219 ± 0.6626
	$K_{{\left[\text{DHIV}\right]}_{1}}^{\text{M}}$	*c*_2 _· $K_{{\left[{\text{NADP}}^{\text{+}}\right]}_{1}}^{\text{M}}$	
	$k_{\text{+3}}^{\text{cat}}$	*c*_3 _· $K_{{\text{[KIV]}}_{\text{1}}}^{\text{M}}$	
	$K_{{\text{[KIC]}}_{\text{2}}}^{\text{M}}$	*c*_4 _· $K_{\text{[KIV]}}^{\text{M}}$ + *c*_5 _· $K_{{\text{[Glut]}}_{\text{2}}}^{\text{M}}$	

The table lists the parameters that are replaced by linear regression models with coefficients *a*_*i*_, *b*_*i*_, *c*_*i *_of other highly correlated model parameters.

## Conclusion

The purpose of this study is to identify both the most suitable modeling approach and the best-performing optimization algorithm to calibrate the parameters contained in metabolic network models. To this end, we constructed one probabilistic and six deterministic mathematical descriptions of valine and leucine biosynthesis in *C. glutamicum*. The parameters of each model were optimized with respect to *in vivo *measurements for the reacting species within the system. In this way we compared eight optimization procedures. We systematically benchmarked both the algorithms and the alternative models to highlight their advantages and drawbacks. In the following paragraphs, we draw several conclusions from the comparison of these seven variants of a realistic reaction system, and we assume them to hold for similar systems. Thus, if no prior knowledge about a comparable metabolic system is available, our results can serve as a starting point for model construction and calibration.

Let us consider the capabilities of the modeling approaches in more detail, when taking into account the ability to approximate measured data, the hybrid model for the reversible system based on convenience rate laws and Michaelis-Menten equations (CKMMr) has the best performance. At the same time, this is the most complex model with respect to the number of parameters and computational costs for each simulation. The acceptable parameter values for this model, found by multiple optimization runs, varied over several orders of magnitude. This corresponds to the fact that the optimization problem shows a large number of local optima, which is often referred to as multimodal behavior. Furthermore, this model is integrable with parameter values selected by chance from an almost arbitrarily wide range. This means that no preliminary data analysis in enzyme databases is required to obtain an integrable start population for the CKMMr model.

On the other hand, a simplified deterministic description of the reaction system based on the generalized mass action rate law (GMAKr) yields good performance as well. Its advantage over the CKMMr model is its small number of parameters. Its major disadvantage is the strong tendency to become non-integrable when selecting parameter values by chance from a larger range. A restriction of the parameter space is required to ensure numerical stability when integrating metabolic network models. Some of the parameters showed almost no variance among the results of multiple optimization runs.

For models of biochemical systems with low metabolic concentrations or systems operating close to the point of instability, stochastic effects should be considered. Generally, simulating large stochastic models is computationally not feasible. The Langevin approach is a simplified stochastic description which facilitates taking these effects into account at acceptable computational costs. For the specific biochemical example network considered in this study, the stochastic effects are negligible as the behavior of the Langevin model is similar to the GMAKr model due to the rather large molecular populations. However, the benchmark showed that this approach is suitable for large-scale parameter optimization and model inference.

Modeling certain reactions in a non-reversible way as was done in all remaining models leads to a significantly worse ability to fit the measured data. We conclude that possible side effects are compensated by means of the reverse reaction. When modeling multi-enzyme systems all reactions should be treated reversibly, unless there is significant biological evidence to introduce irreversible rate laws. If neither the kinetic equations nor meaningful ranges of the parameter space are known, the model should be constructed using convenience kinetics. If the parameter space can be restricted using prior knowledge and the number of parameters matters, a model based on generalized mass-action rate laws constitutes an appropriate choice.

The second aim of this study is to identify the best-performing optimization algorithm for parameter estimation. The ability to find good local optima for the parameter values is the first quality measure for the algorithms. All five evolutionary algorithms tested yielded reasonable performance. From a user perspective, these algorithms differ in the number of settings which influence their behavior and are therefore more or less easy to apply. Hence, the effort to find a good configuration for an algorithm constitutes the second criterion of quality. The Tribes algorithm was among the best-performing algorithms in our benchmarks. As a settings-free optimization procedure, it is the most user-friendly method. However, other algorithms are able to yield even better results after fine-tuning. Particularly, DE and PSO provided the best performance while keeping the effort necessary for their fine-tuning within reasonable limits. ES and binGA are also able to identify valuable local optima for all systems but require examining a large number of well-established alternative operators for their crossover and mutation steps.

For first optimization attempts, the easy to use Tribes algorithm is a good choice. With slightly more effort, the user can adjust the algorithms PSO and DE to yield even better results. Combined with the convenience kinetics modeling approach, these algorithms provide a suitable choice to model unknown systems of metabolic reactions.

## Methods

### The biochemical example network

Figure 1 illustrates the biosynthesis of Val and Leu in a process diagram [68,69] according to the METACYC[8] and KEGG[6,7] databases. The pathway starts with pyruvate (Pyr), from which Val and Leu are produced. Both products are used for biomass production or can be transported out of the cell if not needed. It is important to note that this pathway is regulated by both products in six feedback inhibition mechanisms. The transport of Leu and Val across the cell wall is actually performed by the same enzyme, so that both substrates compete with each other. However, for modeling purposes two distinct reactions are necessary in which the competition is included as inhibition. Some reactions are lumped together (Table 1) as suggested by Magnus *et al*. [52]. Since the reaction 2 IPM) ⇌ 3 IPM is fast, it is assumed to be in equilibrium. This and the two following reactions 3 IPM + NAD^+ ^→ 2 I_3_OS + NADH_2 _as well as (2S)-2-isopropyl-3-oxosuccinate (2 I_3_OS) → 2-ketoisocaproate (KIC) +CO_2 _that only depend on the concentration of 2 IPM are lumped together, introducing the symbol IPM for both derivatives.

### Glucose stimulus-response experiment

After a 10 min starvation period, a glucose pulse was added to the culture medium increasing the glucose concentration from 0 to 3.5 g/l. This glucose step-function induced a dynamic response from the metabolic intermediates linked to this central nutrient. Over a time span of 25 s, beginning 4 s before the glucose pulse, 47 samples were taken for 13 metabolites on the pathway starting at the state of Pyr, which is generated during phosphotransferase system-mediated glucose uptake and is also the final product of glycolysis. Immediate quenching and cooling with methanol to -30°C prevented the metabolites from further reactions. Mass spectrometry (HPLC MS/MS) was used to quantify the metabolite concentrations in the probes. For details of this experiment we refer to Magnus *et al*. [52]. For technical reasons, NADH_2 _and NADPH_2 _as well as acetylCoA and CoA could not be measured with a high degree of exactness. Thus, Magnus *et al*. [52] suggested taking into account that both couples, NAD^+ ^and NADH_2 _as well as NADP^+ ^and NADPH_2_, follow a conservation relation so that the total amount of both coupled metabolites remains constant during the 25 s of interest. Thus, NADH_2 _= 0.8 mM - [NAD^+^] and [NADPH_2_] = 0.04 mM - [NADP^+^]. We assume a constant pool of the other two central metabolites that does not vary over the considered time span. The steady-state concentrations (Table 6) of the seven metabolites to be simulated serve as initial values for the models.

**Table 6:** Steady-state concentrations of the reacting species

Metabolite	Concentration (mM)
AcLac	0.236
DHIV	0.132
IPM	0.0227
KIC	0.0741
Leu	0.209
Val	29.4

The data in this table serve as the initial values of all differential equation systems derived herein.

### Mathematical models

The rates of change of each metabolite's concentration over time can be calculated by linear combination of the stoichiometric matrix **N **describing the structure of the reaction system, i. e., its topology, with the vector of reaction velocities **v **that depends on the vector of reacting species **S**, the parameter vector **p**, and may also explicitly depend on time *t*:

(1) $$\frac{\text{d}}{\text{d}t}S=Nv(S(t),t,p).$$

For the resulting seven-dimensional differential equation system [see Additional file 2]. The databases KEGG[6,7] and METACYC[8] do not indicate if the reaction network pictured in Figure 1 contains irreversible reactions besides the draining of both products as listed in Table 1. One way to study the influence of the existence or non-existence of reverse reactions on the dynamics of the whole system is to derive alternative models and investigate their ability to approximate the data. The simpler irreversible reactions are favored if they are able to fit the data. The following paragraphs present the general equations for all rate laws, which are inserted for each *v*_*i *_and investigated in this study. For the resulting differential equation systems [see Additional file 2]. An implementation of the fourth-order Runge-Kutta method [70] solves the ordinary differential equation systems. The stochastic Langevin system is adapted to be integrated using the MATLAB™ integrator ode15s [71,72].

#### Generalized Mass Action Kinetics (GMAKr)

The simplest rate law is mass action kinetics, in which the effects of the participating enzymes are hidden in the rate constants. To include inhibition effects, we apply an inhibition function that fits the generalized mass action rate law [5,73]:

(2) $$v_{j}(S,p)=F_{j}(S,p)\left(k_{+j}\prod_{i}S_{i}^{n_{ij}^{-}}-k_{-j}\prod_{i}S_{i}^{n_{ij}^{+}}\right).$$

The function *F*_*j*_(**S**, **p**) must be a positive function of the substrate concentrations **S **and the parameter vector **p **to introduce saturation or inhibition effects to the common mass action rate law written in brackets [5]. For convenience of notation, the matrices **N**^± ^are introduced, whose elements $n_{ij}^{\pm }$ express the absolute values of the positive or negative stoichiometric coefficients. Feedback inhibition loops are included using the following approaches:

(3) $$F_{j}(S,p)=\frac{1}{1+K_{j}^{\text{I}}\cdot [\text{I}]}$$

(4) $$F_{j}(S,p)=\exp(-K_{j}^{\text{I}}\cdot [\text{I}])$$

with $K_{j}^{\text{I}}⩾0$. While Equation (4) is derived intuitively, driven by the assumption that the exponential function constitutes an important growth and shrinkage function in biology, Equation (3) can be derived from the competing reactions of the enzyme with its substrate or inhibitor. The first equation can be derived using the equilibrium constant for the inhibition reaction and the conservation law of the enzyme as well as the enzyme-inhibitor complex concentrations. Applying Equation (2) combined with Equation (3) to reaction system *R*_1 _through *R*_10 _leads to an Ordinary Differential Equation (ODE) system with 24 parameters *k*_± *j*_, $K_{j}^{\text{I}}$.

#### Irreversible GMAK with exp inhibition (GMAKi)

By setting all product concentrations apart from *R*_2 _and *R*_9 _to zero and applying Equation (4) to Equation (2) we obtain the irreversible version of this equation system with 18 parameters *k*_± *j*_, $K_{j}^{\text{I}}$.

#### Michaelis-Menten equations (GMMr)

Three reactions of the system (*R*_3_, *R*_6_, and *R*_10_, Table 1) follow a Michaelis-Menten mechanism. The corresponding rate law is given by Equation (5), where S forms product P and the catalyst E is inhibited by I:

(5) $$v_{j}=\frac{\frac{v_{+}^{\text{m}}}{K_{\text{S}}^{\text{M}}}[\text{S}]-\frac{v_{-}^{\text{m}}}{K_{\text{P}}^{\text{M}}}[\text{P}]}{1+\frac{\left[\text{I}\right]}{K^{\text{I}a}}+\left(\frac{\left[\text{S}\right]}{K_{\text{S}}^{\text{M}}}+\frac{\left[\text{P}\right]}{K_{\text{P}}^{\text{M}}}\right)\left(1+\frac{\left[\text{I}\right]}{K^{\text{I}b}}\right)}.$$

In the case of *R*_3 _there might be a reverse reaction. *R*_6 _and *R*_10 _are assumed to be irreversible because they describe the transport of Val and Leu out of the cell. We further assume that the constants *v*^m ^in *R*_6 _and *R*_10 _are allowed to be zero so that there is no need to export Val or Leu if it is needed for biomass formation. All other reactions are modeled using the GMAKr approach including Equation (3) for inhibition. The complete GMMr model contains 31 parameters to be estimated. To avoid numerical problems, the inhibition constants in Michaelis-Menten kinetics are transformed into their reciprocals KIa|b'=1KIa|b. This modification allows the optimization procedure to "decide" which kind of inhibition occurs [5] [see Additional file 2].

#### Irreversible Michaelis-Menten Model (GMMi)

From the GMMr model an irreversible alternative is established by setting all product concentrations to zero. The resulting system contains 24 parameters $K_{j}^{\text{Ia}|\text{b}}$, *k*_± *j*_, $K_{ij}^{\text{M}}$ to be estimated.

#### Reversible Convenience Kinetics (CKMMr)

The general equation of the convenience rate law for reaction *j *reads

(6) $$v_{j}=[{\text{E}}_{j}]\prod_{m}h_{\text{A}}{\left(S_{m},K_{jm}^{\text{A}}\right)}^{w_{jm}^{+}}h_{\text{I}}{\left(S_{m},K_{jm}^{\text{I}}\right)}^{w_{jm}^{-}}\cdot \frac{k_{+j}^{\text{cat}}\prod_{i}{\left(\frac{S_{i}}{K_{ji}^{\text{M}}}\right)}^{n_{ij}^{-}}-k_{-j}^{\text{cat}}\prod_{i}{\left(\frac{S_{i}}{K_{ji}^{\text{M}}}\right)}^{n_{ij}^{+}}}{\prod_{i}\sum_{m=0}^{n_{ij}^{-}}{\left(\frac{S_{i}}{K_{ji}^{\text{M}}}\right)}^{m}+\prod_{i}\sum_{m=0}^{n_{ij}^{+}}{\left(\frac{S_{i}}{K_{ji}^{\text{M}}}\right)}^{m}-1}$$

with *h*_A _and *h*_I _being functions for activation or inhibition, respectively, the turnover rates $k_{\pm j}^{\text{cat}}$ and the matrices **W**^± ^containing positive entries for the connectivity of the modulating metabolites as well as $K_{ji}^{\text{M}}$ being a constant analogous to the Michaelis constant *K*^M ^[16]. For inhibition, which plays an important role in Val and Leu biosynthesis of *C. glutamicum*,

(7) hI(Si,KI)=KIKI+Si=11+SiKI=11+KI'Si

has been suggested and herein applied. Besides the reciprocal constant this approach equals Equation (3). The product [E_*j*_]$k_{\pm j}^{\text{cat}}$ is lumped into one parameter $V_{\pm j}^{\text{m}}$ for all *j *assuming that all enzyme concentrations remain constant during the 25 s. No enzyme concentrations have been measured, so that an optimizer cannot distinguish between the product of two parameters or one parameter. The three reactions that follow the Michaelis-Menten mechanism are modeled using Equation (5). The reactions *R*_6 _and *R*_10 _are considered irreversible as described before. Applying Equation (6) to all remaining reactions in the system *R*_1 _through *R*_10 _yields an equation system with 59 parameters. The stoichiometric matrix has full column rank. Hence, the parameters $k_{\pm j}^{\text{cat}}$ can be estimated directly without violating thermodynamic constraints [16].

#### Irreversible Convenience Kinetics (CKMMi)

By setting all product concentrations apart from *R*_2 _and *R*_9 _to zero, we obtain an irreversible version of this model containing 41 parameters.

#### Stochastic modeling based on the Langevin equation (LANG)

The concentration variables *S*_*i *_are replaced by the random variables *X*_*i*_(*t*) ≡ number of *S*_*i *_molecules in the system at time *t*, *i *= 1,...,*N *in an enclosing reaction volume *V*, where the *N *species interact through *M *reaction channels *R*_*j*_, *j *= 1,...,*M*. Each reaction is characterized by a stochastic rate constant *c*_*j *_depending only on the physical properties of the reacting molecules [11]. In the case of large systems with high metabolite concentration these simulation strategies are highly computationally intensive and therefore unsuited for large-scale parameter optimization. However, for macroscopic systems it is possible to directly approximate the time evolution of the stochastic state variables by the chemical Langevin equation [10,11], which reads

(8) $$\text{d}x_{i}(t)=\sum_{j=1}^{M}n_{ij}a_{j}(x(t))+\sum_{j=1}^{M}n_{ij}\sqrt{a_{j}(x(t))}\text{d}W_{j},\;i=1,...,N$$

when rewritten using the Wiener process [74] for easier numerical treatment. Here *n*_*ij *_represents the stoichiometric coefficient of the *i*^th ^metabolite in the *j*^th ^reaction. The propensity *a*_*j *_is defined as: *a*_*j*_d*t *= *c*_*j*_*h*_*j*_d*t *≡ probability that an *R*_*j *_reaction will occur in *V *in (*t, t *+ d*t*), given that the system is in state (*X*_1_,...,*X*_*N*_) at time *t*. The function *h*_*j *_gives the number of distinct *R*_*j *_molecular reactant combinations available in the state (*X*_1_,...,*X*_*N*_), *j *= 1,...,*M*. The discrete variables (*X*_1_,...,*X*_*N*_) are replaced by the continuous molecule concentrations (*x*_1_,...,*x*_*N*_). In order to numerically integrate the Langevin equation with standard ODE solvers, the equation is split into a stochastic and a deterministic term. The deterministic term and the deterministic part of the stochastic term are treated like ODEs, as suggested by Bentele *et al*. [75]:

(9) $$\Delta {\hat{x}}_{i}(t)=\sum_{j=1}^{M}n_{ij}a_{j}(x_{t})\Delta t$$

(10) $$\Delta {\tilde{x}}_{i}(t)=\sum_{j=1}^{M}n_{ij}\sqrt{a_{j}(x(t))\Delta t}.$$

The latter term is then multiplied by a normal random variable *n*_*i *_= N in analogy to the finite Wiener increments used in the Euler-Maruyama method [74]. After each time-step, both terms are added to give the full state-variable change:

(11) $$\Delta x_{i}(t)=\Delta {\hat{x}}_{i}(t)+\Delta {\tilde{x}}_{i}(t)\cdot n_{i}$$

allowing adaptive step-size control of the ODE solver. The reaction propensities are calculated according to Gillespie [11]. This leads to an equation system with 24 parameters.

#### Representing external metabolites with splines

As suggested by Magnus *et al*. [52], metabolites whose concentrations cannot be explained in terms of the model are considered external, i. e., they are an input to the model but involved in numerous other reactions that are not reflected by this system (Figure 1). We approximate these using cubic splines which smooth the measurements. To weight all measurements equally, all *ω*_*i *_are set to 1. Due to the different ranges of the concentrations of the six metabolites, it is not possible to find one appropriate degree of smoothness *λ *that leads to equally smooth curves. Hence, we transform all concentrations into the range [0, 1], set *λ *= 1, compute the spline coefficients and re-transform them back into the original range of the specific metabolite. For details and a figure showing the resulting splines [see Additional file 2].

### Fitness function and search-space restrictions

Since many distance measurements have been defined, the choice of the most appropriate one is an important step for the parameter estimation process. Due to the differences in the concentrations of certain metabolites the Euclidian distance between the model values and the measurements is not applicable: metabolites in higher concentration would dominate in fitness over those in lower concentration. Minimizing the relative squared error (RSE, see Equation (12)) overcomes this limitation. The outer sum runs over all dimensions of $\hat{x}$ describing the model output at each sample time *τ*_*t*_. *T *is the number of measurements taken and **X **= (*x*_*ti*_) is the given data matrix.

(12) $$f_{\text{RSE}}(\hat{x},X)=\sum_{i=1}^{\dim(\hat{x})}\sum_{t=1}^{T}{\left(\frac{{\hat{x}}_{i}(\tau_{t})-x_{ti}}{x_{ti}}\right)}^{2}.$$

This fitness function was used in several publications for similar problems [33,47,48].

To restrict the search space for the optimizers, we limit parameter values to the range [0, 2000], covering 98.748% of all known kinetic parameters in BRENDA [24-26], as suggested in [47,48]. All known parameters in BRENDA are greater than or equal to zero. To avoid division by zero in some parameters, the range is set to [*ε*, 2000] with *ε *= 10^-8^. For parameters transformed in Michaelis-Menten equations (as described above), the range is limited to [0, 10^8^], resulting in a search space from 10^-8 ^through ∞. Only 0.962% of all *K*^I ^and 0.004% of all *K*^M^values in BRENDA are reported to be lower than 10^-8^. This *ε *is chosen to guarantee numerical stability. These restrictions are applied to all parameters. In cases where no division by the parameter value is necessary, *ε *= 0 is allowed to be a lower bound of the parameter range.

All parameters are initialized with low values to avoid obtaining unstable initial populations and it is assumed that large parameter values are rather infrequent in nature [47,48]. A Gaussian distribution with *μ *= *σ *= 1 guarantees low initial values and ensures stable initial populations. Each parameter is set to the boundary values if it breaks any of the search-space restrictions. We limit the initialization procedure for all models to a low value initialization to ensure equal conditions in all comparisons.

### Systematically improving the performance of the optimization procedures

#### Standard settings for the optimization algorithms

For a comprehensive introduction to all optimization algorithms used in this study [see Additional file 1], in which we also explain the specific settings and operators for each method in detail.

Using the open-source framework EVA2 for nature-inspired optimization procedures [37,38], we test the following standard settings of the algorithms (Table 2) on the inference problem, of which the following are evolutionary optimization procedures:

• Binary Genetic Algorithm (binGA) with one-point mutation, *p*_*m *_= 0.1, and one-point crossover, *p*_*c *_= 0.7.

• Real-valued Genetic Algorithm (realGA) with global mutation, *p*_*m *_= 0.1, and UNDX crossover, *p*_*c *_= 0.8. Both GAs use tournament selection with a group size of 8 in a population of 250 individuals.

• Standard Evolution Strategy (stdES) as (5,25)-ES with global mutation, *p*_*m *_= 0.8, and discrete one-point crossover, *p*_*c *_= 0.2.

• Evolution Strategy with covariance matrix adaption with and without elitism, i. e., "plus" strategy, (cmaES/cmaESplus) with *μ *= 5 and *λ *= 25, *p*_*m *_= 1.0, and no crossover. All ESs use deterministic best-first selection to choose the next generation.

• Differential Evolution (DE) with the scheme DE/current-to-best/1 [42] setting *λ *= *F *= 0.8, *CR *= 0.5 and a population size of 100.

Two optimization strategies are swarm intelligence optimization procedures:

• Constricted Particle Swarm Optimization (PSO) [45,76]: setting *ϕ*_1 _= 2.05, *ϕ*_2 _= 2.05, *χ *= 0.73 using star topology and a population size of 100 as well as its derivative

• Tribes [46]

We also study the following classical non-evolutionary methods:

• Monte Carlo Optimization (MCO) with 50 multi-runs

• (Multi-start) Hill Climber (HC), the number of starts varying from 1, 10, 25, 50, 100 to 250 using Gaussian mutation with a fixed standard deviation of *σ *= 0.2 and a mutation probability *p*_*m *_= 1.0.

• Simulated Annealing (SA) with *α *= 0.1 and an initial temperature of *T *= 5 using a linear annealing schedule and a population size of 250.

For all algorithms with population sizes lower than 250 individuals, a pre-population with 250 parameter vectors is generated and the best are selected to create the initial population. This step is crucial to obtain comparable results for algorithms with different population sizes [33]. Every setting described in this and all following paragraphs is repeated 20 times with 100,000 fitness evaluations per run on the deterministic models. The results of this preliminary trial are shown in Table 3 and Figure 3. The methods found to be successful in these analyses are utilized to optimize the Langevin model as well.

#### Alternative settings for the best optimization algorithms

We first study the influence of various mutation and crossover operators on the three deterministic reversible models for binGA and ES. In a grid search, all combinations of the following operators are evaluated, and we exclude the combinations of no crossover with no mutation. For binGA, no mutation, one-point, and adaptive mutation, an operator which modifies individual mutation probabilities similar to ES step-size adaptation, is tested, paired with one- and *n*-point (*n *= 3), uniform, and bit-simulated crossover, each with *p*_*m *_= 0.1, *p*_*c *_= 0.7. On ES we use the mutation operators covariance matrix adaptation (CMA), the 1/5^th ^success rule as well as correlated, global, local, and no mutation paired with the crossover operators one- and *n*-point (*n *= 3), UNDX, and no crossover, each with *p*_*m *_= 0.8, *p*_*c *_= 0.2. To study the influence of the mutation or the crossover operator alone, the values for *p*_*m *_and *p*_*c *_are set to zero or one, depending on which influence is investigated. The population size for binGA is set to 100 and for ES (5, 25) (Figure 4). For the most successful operator combination (adaptive mutation with bit-simulated crossover) on the GMAKr model, we study the influence of the probabilities *p*_*m *_and *p*_*c*_, with which the respective operator is invoked by binGA. All pairs of *pm *and *pc *are evaluated from 0.0 through 1.0 in 0.1 steps and a population size of 100, excluding *p*_*m *_= *p*_*c *_= 0.0 (Figure 5(a)). Subsequently, the population sizes 50, 100, 250, 500, 1,000, and 2,000 are tested for binGA with *p*_*m *_= 0.2 and *p*_*c *_= 1.0 (Figure 5(b)). For the cmaESplus, we evaluate all combinations of *μ *∈ {5, 10, 25, 50, 75}, and *λ *∈ {10, 25, 50, 75, 100, 125, 150}, excluding cases where *μ *> *λ *and keeping *p*_*m *_at 1.0, and *p*_*c *_at 0.0 (Figure 6).

For the DE approach, another grid search is performed on the three reversible deterministic models, altogether testing values for *F*, *λ *∈ {0.5, 0.8}, and *CR *∈ {0.3, 0.5, 0.9} (Figure 7). For the most promising parameter set (*F *= 0.8, *λ *= 0.5, and *CR *= 0.3 for GMAKr and GMMr, and *CR *= 0.9 for the CKMMr), the population size is additionally varied over {50, 250, 500, 1000} for each model (Figure 8).

The PSO with star topology and standard settings for *ϕ*_1 _= *ϕ*_2 _= 2.05 is compared with a star topology and settings *ϕ*_1 _= 2.8, *ϕ*_2 _= 1.3, as suggested in [77] as well as a linear 3 and grid 3 topology with standard parameters on all three reversible deterministic models (Figure 9). The population size is set to 25. Additional population sizes, namely {50, 250, 500}, are evaluated using a grid 3 topology and standard values for *ϕ*_1 _and *ϕ*_2 _on these models (Figure 10).

### Parameter-space analysis

The completion of the large-scale parameter optimization study in the first part yields a large set of different optimal parameters. We select all parameter sets with a fitness less than 25 for each reversible deterministic model (GMAKr, GMMr, and CKMMr).

#### Cluster analysis

To cluster these best parameter sets (Figure 12), we apply the agglomerative nesting algorithm (AGNES) [[78], pp. 199–251], using a Euclidean metric, which is implemented in the cluster package [79] of the R-project [80].

#### Variance analysis to visualize the scattering of each parameter

The variances of each parameter among the best optimization results of the three reversible deterministic models are calculated and plotted on a logarithmic scale due to the large differences in their orders of magnitude (Figure 13).

#### Multiple correlation analysis

Multiple correlation *r*_*Y*_,(*X*_1_,...,*X*_*p*_), measures the dependency of one model parameter *Y *on *p *other parameters *X*_1_,...,*X*_*p *_of the model. Here, *r*_*Y*_,(*X*_1_,...,*X*_*p*_) is defined as the largest simple correlation among the correlations of *Y *and all linear combinations of the *X*_*i*_, that is $\sum_{i=1}^{p}a_{i}X_{i}$, with arbitrary weights *a*_*i*_. A large value of *r*_*Y*_,(*X*_1_,...,*X*_*p*_) indicates a strong dependence of *Y *on other model parameters and suggests that *Y *is not a genuine degree of freedom of the model. In order to calculate the multiple correlation of *Y *and *X*_1_,...,*X*_*p *_within a sample of size *n *different runs for each parameter, all simple correlations *r*_*Y*_, *X*_*i *_must be determined using the Pearson correlation coefficient *r*_*Y*_, *X*_*i *_[81]:

(13) $$r_{Y,X_{i}}=\frac{\sum_{i=1}^{n}x_{i}y_{i}-n\bar{x}\bar{y}}{\sqrt{\left(\sum_{i=1}^{n}x_{i}^{2}-n{\bar{x}}^{2}\right)\left(\sum_{i=1}^{n}y_{i}^{2}-n{\bar{y}}^{2}\right)}}.$$

A correlation of ± 1 means that there is a perfect positive/negative linear relationship between the parameters *Y *and *X*. In that case, the parameter *Y *can be explained by parameter *X *and therefore be omitted. The multiple correlation is then established from all of these simple correlations [81]:

(14) $$r_{Y,(X_{1},...,X_{p})}=\sqrt{r_{YX}^{T}R_{\left(XX\right)}^{-1}r_{YX}}={\left([r_{YX_{1}},...,r_{Y,X_{p}}]{\left[\begin{array}{cccc} 1 & r_{X_{1}X_{2}} & \cdots & r_{X_{1}X_{p}}\\ r_{X_{1}X_{2}} & 1 & \cdots & r_{X_{2}X_{p}}\\ \vdots & \vdots & \ddots & \vdots \\ r_{X_{1}X_{p}} & r_{X_{2}X_{p}} & \cdots & 1 \end{array}\right]}^{-1}\left[\begin{array}{c} r_{YX_{1}}\\ r_{YX_{2}}\\ \vdots \\ r_{YX_{P}} \end{array}\right]\right)}^{\frac{1}{2}}.$$

The coefficient of determination

(15) $$B=r_{Y,(X_{1},...,X_{p})}^{2}$$

measures how well the paremeter *Y *can be explained in a linear sense by the other model parameters *X*_1_,...,*X*_*p*_. After identification of highly correlated parameters, a subset of replacement candidates is selected. A parameter *X *is considered a replacement candidate for parameter *Y *if *r*_*X*,*Y *_≥ 0.7 and the *p*-value of the correlation, computed using a *t*-test, is close to zero. The degrees of freedom selected for replacement are subsequently substituted by a linear regression model of their correlated parameters.

### Hardware configuration

All experiments are run on a cluster with 16 AMD dual Opteron CPUs with 2.4 GHz, 1 MB level 2 cache, and 2 GB RAM per node under the Sun Grid Engine, and JVM 1.5.0 with Scientific Linux 5 as operating system. An optimization of one model in 20 parallel multi-runs takes approximately 1.5 h.

### Availability of models and optimization procedures

All models investigated in this study are included as optimization problems in EvA2, a Java™-based workbench for heuristic optimization [37,38], which can be downloaded at .

## Authors' contributions

AD developed the conceptual idea, created all deterministic models, and wrote Additional file 2 together with MJZ. MK is the main developer of EVA2 and performed the optimization parts of this study together with AD. MK selected and implemented the algorithms to be tested, suggested alternative settings for the optimizers, and wrote Additional file 1. MJZ created and optimized the stochastic model, and performed all parameter space analyses. AD, MK, MJZ, and JS wrote this manuscript. HP implemented the integrator and parts of EVA2, and supported the optimization. JM and MO performed the *in vivo *measurements for the model system. OK and AZ supervised the work. All authors read and approved the final manuscript.

## Supplementary Material

Additional file 1 **Nature-inspired heuristics for optimization**. This document provides a detailed introduction to the basic concepts of all evolutionary and swarm intelligence algorithms that are used in this study. Click here for file

Additional file 2 **Modeling the valine and leucine metabolism in *Corynebacterium glutamicum***. This document gives a comprehensive introduction to mathematical modeling of biochemical systems and all four kinds of rate laws that are used in this study. Additionally, the method to introduce inhibition effects to the generalized mass action rate law is derived in this document. Histograms are presented showing the distribution of the parameters. For the best-performing optimization algorithms, the fitness is plotted depending on the evaluations of the objective function. Click here for file
