# Supplementary material for: Modeling metabolic networks in C. glutamicum: a comparison of rate laws in combination with various parameter optimization strategies
Source: BMC Syst Biol. 2009 Jan 14;3:5. doi: 10.1186/1752-0509-3-5 (PMC2661887; doi:10.1186/1752-0509-3-5)
Supplement: Additional file 1 — Nature-inspired heuristics for optimization. This document provides a detailed introduction to the basic concepts of all evolutionary and swarm intelligence algorithms that are used in this study. [file 1752-0509-3-5-S1.pdf]

EBERHARD-KARLS-UNIVERSITÄT TÜBINGEN
Wilhelm-Schickard-Institut für Informatik
Lehrstuhl Rechnerarchitektur

Additional file 1

Nature-inspired heuristics for optimization

Marcel Kronfeld* Andreas Dräger Michael Ziller
Jochen Supper Hannes Planatscher Jørgen B. Magnus
Marco Oldiges Oliver Kohlbacher Andreas Zell

January 9, 2009

*Corresponding author, e-mail: marcel.kronfeld@uni-tuebingen.de

Contents

1 Introduction **5**

2 Selected Non-Evolutionary Algorithms **5**
2.1 Hill Climber 5
2.2 Simulated Annealing 5

3 Evolutionary Algorithms **6**
3.1 Genetic Algorithm 6
3.2 Evolution Strategy 7
3.3 Differential Evolution 8

4 Swarm Intelligence Algorithms **9**
4.1 Particle Swarm Optimization 9
4.2 Tribes: a Parameter-free PSO 9

References **10**

1 Introduction

Optimization or, without loss of generality, minimization, may be defined as the search for a vector x_0 in a possible solution set X minimizing a target function f so that $\forall x \in U \subseteq X: f(x) \geq f(x_0)$. For $U = X$, x_0 is called a *global optimum*, otherwise it is called a *local optimum* of f in X.

A heuristic optimization method tries to find the global optimum of a function which is analytically hard or infeasible to solve because, among other reasons, it is non-convex with numerous local optima and the gradient cannot be easily computed.

Simple classical optimization methods iteratively evaluate one solution and try to improve it until a local optimum is found. Among these, we will introduce the hill climber [1] and simulated annealing [2] in Section 2. Many modern heuristics have been inspired by natural analogies and iteratively search the solution "intelligently" using a natural concept. They are usually population-based and evaluate several possible solutions in parallel during an iteration, thus using more information and increasing the possibility of escaping local optima. Among the most widespread natural analogies are the Darwinian evolution principle and the swarm intelligence idea, which we want to introduce together with their most important parameter settings[1] in Sections 3 and 4.

2 Selected Non-Evolutionary Algorithms

2.1 Hill Climber

The Hill Climber (HC) produces an initial solution x_{t_0}, then scans a small environment U_{t_0} of that solution and sets x_{t_1} to the best solution of U_{t_0}. By repeating this step, the current solution is improved until a local optimum in U_{t^*} is reached, much like a mountaineer cresting a hill. Both solution quality and run-time of the algorithm greatly depend on the size of U_t. Usually, U_t must be small to make the computation feasible, which leads to the probability of finding the global optimum among many local optima to be rather small. Therefore, a multi-start HC is restarted several times at different starting points to increase this probability.

2.2 Simulated Annealing

A related technique is called Simulated Annealing (SA). SA was inspired by the annealing processes in metallurgy, where slow cooling improves the crystal structures of materials. It uses a "temperature" parameter T to determine whether a solution is accepted as new x_{t+1} at a time $t+1$, even if it has a worse fitness value than x_t. Specifically, for the case of maximization, x_{t+1} is accepted with probability $\exp\left(\frac{1}{T}\left[f(x_{t+1}) - f(x_t)\right]\right)$ if $f(x_{t+1}) < f(x_t)$. T is decreased with time by multiplying it with a parameter $\alpha < 1$, which allows the algorithm to escape local optima with some probability at the beginning of a run but makes it converge on a local optimum at the end.

[1] Note that these strategy parameters are settings of the respective algorithm and therefore to be distinguished from parameters to be estimated in an optimization problem.

3 Evolutionary Algorithms

The Darwinian theory of evolution may be interpreted as a search algorithm working on *individuals* defined by their *genetic representations* which are propagated to the next generation if they achieve a certain *fitness* defined through the probability of survival, finally resulting in a new generation of—possibly—improved individuals. This natural evolution scheme has been translated into an artificial optimization method for computational target functions usually termed an *Evolutionary Algorithm* (EA). EAs consist of the following components:

Representation: Find a way to represent a possible solution (an individual) of the target function.

Initialization: Initialize a set of possible solutions (the population), randomly or using systematic knowledge.

Fitness evaluation: Assemble a fitness function which assigns a quality value with respect to the target function to each possible solution.

Selection: Employ a selection scheme which, from a set of evaluated individuals, selects a promising subset, i. e., a parent population.

Reproduction and variation: From the parent population, create a new population by combining and mutating the parent individuals.

By iterating the evaluation, selection, reproduction and variation steps, it is expected that the individuals in the population improve over time with respect to the target function. This is because of the selection pressure towards better fitness and the property of the offspring containing recombined parts of high quality parents, while still new elements may be introduced through the mutation operator. EAs have been shown to work well under difficult circumstances and are one method of choice if, for example, gradient methods cannot be applied, the target function is noisy and highly nonlinear, and simpler hill climbers fail because of the target function having multiple local optima. Due to the population-based character of an EA, it is able to search larger parts of the search space in parallel. If, in addition to that, the selection scheme is probabilistic or the mutation scheme covers a large subspace with low probability, an EA is able to overcome local optima and find the global optimum with a reasonably high probability. There are mainly two branches of EAs of interest here, developed independently of each other in the 1960s and 70s, and which are described in the following subsections.

3.1 Genetic Algorithm

Genetic Algorithms (GA) became popular through the work of Holland (1975, [3]). His prime idea was to simulate genetic processes in a computer, and in analogy to the four base pairs in organic DNA, he used the discrete counterpart in computation, i. e., binary strings containing zeros and ones, to represent individuals. In order to solve continuous functions, the binary genotype in GA often has to be translated to a continuous phenotype which can be evaluated. The selection scheme is usually implemented in a probabilistic way, giving an individual I of

population P a chance of survival which is proportional to its relative fitness $f_{rel}(I)$ (stated for maximization):

$$f_{rel}(I) = \frac{f(I)}{\sum_{J \in P} f(J)}.$$

Reproduction is done by just copying the parents to a new population of the same size, while recombination works by cutting two randomly chosen parents in $n \geq 2$ pieces and exchanging analogous sub-parts to form children (*crossover*). During mutation, each bit of an individual genotype is then flipped with a certain small probability p_m, e. g., $p_m = 0.001$ (Figure 1). The probability of crossover p_c is often set to a value close to 1.

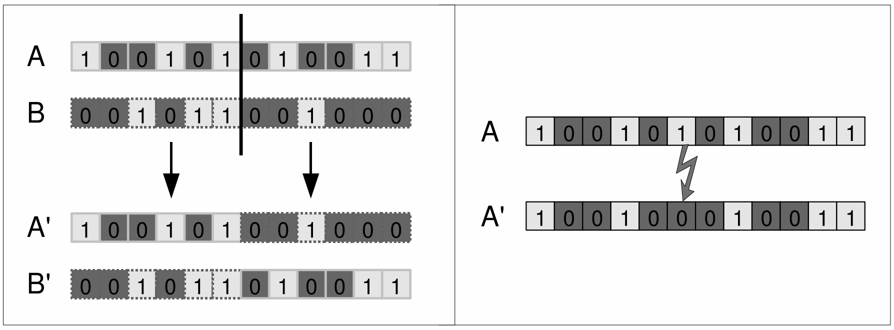

Figure 1: Illustration of crossover and mutation on a binary genotype.

Parameters, which control how the EA works, such as the population size or variation probabilities p_c and p_m are also called strategy parameters of the EA, as opposed to problem parameters making up each single solution representation.

3.2 Evolution Strategy

The second type of EA uses a real-valued genotype and was termed *Evolution Strategy* (ES) by Rechenberg in 1973 [4]. Other than in a GA, the typical ES selection scheme works deterministically, selecting the best μ of the population and copying them to the new population of size $\lambda > \mu$. The offspring are then mutated by adding a small random Gaussian noise $\delta \sim \mathcal{N}(0, \sigma)$, where σ is called the mutation step size. Mutation is done with a rather high probability close to 1, while recombination is typically applied with low probability. After the fitness evaluation, the next generation can be selected from the λ children only, or it may also take the μ last parents into account. The first variant is called (μ, λ)-selection, the second $(\mu + \lambda)$-selection. While the (μ, λ)-scheme may not retain high quality solutions of the parent set, the $(\mu + \lambda)$-scheme has the property that the best individual can never be lost during optimization, because it is always taken into account by the deterministic selection. This can be advantageous, e. g., for a convergence proof, but on the other hand, it may cause the ES to get stuck in local optima, because it is harder to cross “valleys” between optima if an attractor cannot be forgotten.

A possibility developed early in ES is the idea of self-adaption: Strategy parameters can be added to the genotype and be optimized in parallel to the actual problem parameters. This has been proven to be useful especially for the mutation step-size σ, giving each individual the

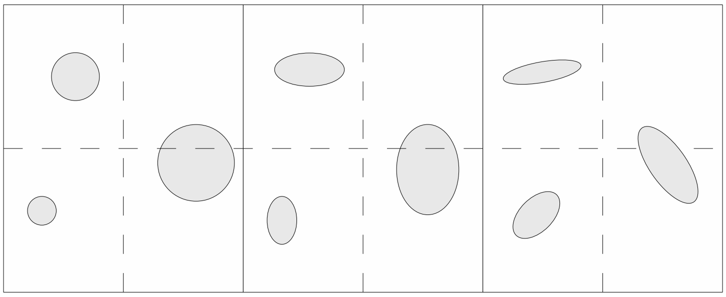

Figure 2: Illustration of ES mutation operators with globally, locally and CMA controlled step sizes.

possibility of finding a preferred step-size for its area of the solution space. Extending the idea, a step-size vector containing $\sigma_i, i \in \{1,\dots,n\}$ parameters may be introduced into each problem dimension n, allowing the mutation to additionally choose preferred axes of mutation parallel to the coordinate system (*local mutation*). A generalization of this is the so-called Covariance Matrix Adaptation (CMA) [5], where an $n \times n$ matrix is added to the individual genotype coding the covariance of the random mutation vector δ. This allows the individuals to discover arbitrary directions in which mutation is especially promising. The CMA-ES variant is widely regarded as most sophisticated and able to solve even hard optimization tasks relatively effectively, as long as n remains rather small, e. g., $n < 100$, because of the quadratic space requirements of the adaptation matrix.

3.3 Differential Evolution

Besides GA and ES, and often inspired by them, several other evolutionary methods have been developed and found to be more or less promising. One of the more successful and interesting approaches of the last several years is called Differential Evolution (DE), presented by Storn 1996 [6]. DE works on real-valued genotypes and defines a variation operator, or rather a class of variation operators, based on selecting parent individuals x_{r1}, x_{r2} and x_{r3} from the population and calculating a difference vector $v = x_C + F(x_A - x_B)$. For each individual x, a new trial individual u is created by selecting the parents and recombining x with v in a crossover step. The amplification parameter $F \in [0,2]$ and the crossover probability CR are strategy parameters. CR is not equivalent to p_c from GA, because CR is interpreted component-wise, meaning that the higher the CR, the more components are taken from v to form u. The trial individual u is then evaluated and replaces x only if its fitness is higher than that of x. In this way, similar to a $(\mu+\lambda)$-ES, DE is unable to "forget" good solutions. Still, because the difference operator is able to create diverse individuals, DE has quite good exploration capabilities. Several suggestions have been made about how to select the parents (at random or by quality) and on the number of difference vectors (usually one or two). Often, DE schemes using a second difference vector towards the best individual for offspring creation (with amplification λ) are superior to random parent selection alone. Typical parameter settings are $\lambda = F = 0.8$ and $CR = 0.5$.

4 Swarm Intelligence Algorithms

4.1 Particle Swarm Optimization

Another class of naturally inspired heuristics uses ideas of *swarm intelligence*. When looking at swarms of fish or birds, for example, one often observes flocking behavior: When deciding on how to move, each individual takes into account not only its own state but the movements of neighboring individuals, so that the collective moves in a seemingly organized way, although there is usually no designated leader of the group. This concept of simple, local interaction producing self-organized behavior has been found to be an interesting approach in artificial intelligence. The corresponding search heuristic is called Particle Swarm Optimization (PSO) and was introduced by Kennedy and Eberhart in 1995 [7]. Similar to EAs, PSO proposes a population of possible solutions which is to be improved iteratively. As opposed to EAs, however, it does not apply evolutionary concepts such as selection, reproduction or crossover. Instead, each individual (or particle) x is thought of as "flying" across the solution space with its own travel velocity v. Also, each particle is assigned to a set of neighboring individuals $N(x)$ defining an overlay topology. During an iteration, the particle updates its velocity using two sources of information. The first one is called the "cognitive component" and draws the particle towards the best position it has itself seen so far on its "voyage". The second one is called "social component" and draws the particle towards the best position which has been found by all of its neighbors. The update formula is implemented componentwise for a vectorial individual x using random variables $r_1, r_2 \sim U(0,1)$ in Equations (1 and 2)

$$v_i(t+1) = \chi[v_t + r_1\phi_1(x_i^p - x_i) + r_2\phi_2(x_i^n - x_i)] \tag{1}$$

$$x_i(t+1) = x_i(t) + v_i(t+1) \tag{2}$$

The random factors r_1 and r_2 allow stochastic exploration. The strategy parameters ϕ_1 and ϕ_2 weigh the historic versus the neighbor-dependent attraction, while the constriction factor χ is smaller than one and can be chosen such that the swarm slowly converges if $\phi_1 + \phi_2 \gtrsim 4$ [8]. Standard settings are $\phi_1 = \phi_2 = 2.05$ and $\chi = 0.73$. As opposed to other EAs, the PSO method is applied with rather small population sizes and shows good exploration abilities. On the other hand, its convergence is often observed to be slower than that of self-adaptive ESs, for example.

4.2 Tribes: a Parameter-free PSO

Whenever there are strategy parameters for a heuristic that influence its behavior, there arises the question of how to set them for a specific case. For most parameters there exist rules of thumb found empirically by systematically testing different settings on collections of benchmark problems. For very specific problems, these settings may not be optimal and it is interesting to find which ones work better and why, to improve optimization performance on the one hand or to improve the optimization method itself on the other.

Still, it is often desirable to just use an out-of-the-box heuristic, employ default parameters without any "meta-optimization" or have them handled by self-adaptive mechanisms. Especially, users beyond the field of computer science, like technicians or biologists, might look for a method without lots of parameters, which can hardly be chosen without knowing details of the

underlying method. This fact was considered by M. Clerc when introducing a PSO variant without any parameters, which he calls Tribes [9]. He combines several mechanisms for choosing a swarm size, swarm topology, initialization and update scheme and argues that, without knowing the optimal setting, it is favorable to mix them randomly and enforce successful ones, similar to self-adaptive parameters in ES but in a more general way. Being interested in how well this works for a biological optimization problem, we added Tribes to the list of candidate optimizers.

References

[1] Tovey CA: **Hill climbing with multiple local optima**. *Alg Disc Meth* 1985, **6**(3):384–393, [`http://link.aip.org/link/?SML/6/384/1`].

[2] Kirkpatrick S, Gelatt CD, Vecchi MP: **Optimization by Simulated Annealing**. *Science* 1983, **220**(4598):671–680, [`http://www.sciencemag.org/cgi/content/abstract/220/4598/671`].

[3] Holland JH: *Adaptation in Natural and Artificial Systems*. The University of Michigan Press 1975.

[4] Rechenberg I: *Evolutionsstrategie: Optimierung technischer Systeme nach Prinzipien der biologischen Evolution*. Fromman-Holzboog, Stuttgart 1973.

[5] Hansen N, Ostermeier A: **Completely Derandomized Self-Adaptation in Evolution Strategies**. *Evolutionary Computation* 2001, **9**(2):159–195.

[6] Storn R: **On the Usage of Differential Evolution for Function Optimization**. In *1996 Biennial Conference of the North American Fuzzy Information Processing Society*, Berkeley: IEEE, New York, USA 1996:519–523.

[7] Kennedy J, Eberhart R: **Particle Swarm Optimization**. In *IEEE Int. Conf. on Neural Networks*, Perth, Australia 1995.

[8] Clerc M, Kennedy J: **The Particle Swarm—Explosion, Stability, and Convergence in a Multidimensional Complex Space**. *IEEE Transactions on Evolutionary Computation* 2002, **6**:58–73.

[9] Clerc M: *Particle Swarm Optimization*. ISTE Ltd 2005.
