# Supplementary material for: Modeling metabolic networks in C. glutamicum: a comparison of rate laws in combination with various parameter optimization strategies
Source: BMC Syst Biol. 2009 Jan 14;3:5. doi: 10.1186/1752-0509-3-5 (PMC2661887; doi:10.1186/1752-0509-3-5)
Supplement: Additional file 2 — Modeling the valine and leucine metabolism in Corynebacterium glutamicum. This document gives a comprehensive introduction to mathematical modeling of biochemical systems and all four kinds of rate laws that are used in this study. Additionally, the method to introduce inhibition effects to the generalized mass action rate law is derived in this document. Histograms are presented showing the distribution of the parameters. For the best-performing optimization algorithms, the fitness is plotted depending on the evaluations of the objective function. [file 1752-0509-3-5-S2.pdf]

EBERHARD-KARLS-UNIVERSITÄT TÜBINGEN
Wilhelm-Schickard-Institut für Informatik
Lehrstuhl Rechnerarchitektur

Additional file 2

Modeling the valine and leucine metabolism in *Corynebacterium glutamicum*

Andreas Dräger* Marcel Kronfeld Michael Johannes Ziller†
Jochen Supper Hannes Planatscher Jørgen Barsett Magnus
Marco Oldiges Oliver Kohlbacher Andreas Zell

January 9, 2009

*Corresponding author, e-mail: andreas.draeger@uni-tuebingen.de
†Corresponding author, e-mail: michael.ziller@student.uni-tuebingen.de

Contents

1 Introduction to Mathematical Modeling of Biochemical Systems 5
1.1 Generalized Mass Action Rate Law 5
1.2 Michaelis-Menten Equation 7
1.3 Convenience Rate Law and Thermodynamics 8
1.4 Stochastic Langevin Equation 9

2 Application to the Valine and Leucine Biosynthesis in *C. glutamicum* 11
2.1 Modeling External Metabolites Using Approximation Splines 13
2.2 Generalized Mass Action Rate Law 14
2.2.1 Reversible Reactions (GMAKr) 14
2.2.2 Irreversible Reactions with exp Inhibition (GMAKi) 15
2.3 Michaelis-Menten Equations 15
2.3.1 Reversible Michaelis-Menten Model (GMMr) 16
2.3.2 Irreversible Michaelis-Menten Model (GMMi) 16
2.4 Convenience Kinetics Model 17
2.4.1 Reversible Convenience Kinetics (CKMMr) 17
2.4.2 Irreversible Convenience Kinetics (CKMMi) 18
2.5 Stochastic Modeling based on the Langevin Equation (LANG) 18
2.6 Optimization of the Model Parameters 19

References 26

1 Introduction to Mathematical Modeling of Biochemical Systems

The topology, of a reaction network can be described with a stoichiometric matrix **N**. Each column in this matrix represents one reaction and each row is assigned to one metabolite, i. e., one reacting species. Negative entries in this matrix imply that a species is consumed whereas positive matrix elements stand for the creation of a species in the associated reaction. To compute the rates of change over time for each species in the system a second quality is required: the velocity of each reaction, i. e., the amount of molecules which are consumed or created per unit time in each reaction. The vector of reaction velocities depends on the vector of reacting species **S**, the parameter vector **p** and may also depend explicitly on time t. If both the structural dependency **N** and the reaction velocities are known, the rates of change of each metabolite's concentration over time can be calculated by linear combination of the stoichiometric matrix **N** with the vector of reaction velocities **v**:

$$\frac{\mathrm{d}}{\mathrm{d}t}\mathbf{S} = \mathbf{N}\mathbf{v}(\mathbf{S}(t), t, \mathbf{p}). \tag{1}$$

In many cases, the stoichiometric matrix of the system is known or can be obtained from on-line databases like KEGG [1, 2] or METACYC [3]. Reliable rate laws for the reactions contained within the network are, however, often unknown because these must actually be derived for each singular reaction by measurements and laborious experiments [4]. Therefore, approximative rate laws are often applied, which are either continuous or discrete and either deterministic or stochastic [5]. Several examples of each group exist such as probabilistic, e. g., the classical Gillespie or the modern Langevin approach [6, 7], phenomenological approaches like power law approximations [8, 9, 10], linlog [11] or loglin [12] kinetics or semi-mechanistic approaches like the generalized mass action rate law [10, 13] or a recent generalization of the Michaelis-Menten equation, the convenience rate law [13]. A different choice of specific rate laws **v** leads to alternative model systems and also to a different parameter vector **p**.

In the remainder of this section we discuss the properties of four often used rate laws. The next section shows the resulting differential equation systems when these equations are applied to the specific system under consideration, the valine and leucine biosynthesis in *C. glutamicum*.

1.1 Generalized Mass Action Rate Law

The mass action rate law is the simplest one because it only contains two parameters: one for the forward and one for the reverse reaction. If the equilibrium constant of the reaction is known, this rate law can be even further simplified. To reduce the model's complexity, it may in some cases be desirable to neglect the effects of an enzyme catalysis. The mechanism can be described by a mass action rate law instead, in which the effects of the participating enzymes are hidden in the rate constants. However, if any kind of inhibition is involved in the reaction, this has to be included in the kinetic equation. Here we apply an inhibition function that fits in the generalized mass action rate law as proposed by Schauer and Heinrich in 1983 [10, 14]:

$$v_j(\mathbf{S}, \mathbf{p}) = F_j(\mathbf{S}, \mathbf{p}) \left(k_{+j} \prod_i S_i^{n_{ij}^-} - k_{-j} \prod_i S_i^{n_{ij}^+} \right). \tag{2}$$

The function $F_j(\mathbf{S},\mathbf{p})$ has been defined as any positive function of the substrate concentrations $\mathbf{S}$ and the parameter vector $\mathbf{p}$ to introduce saturation or inhibition effects to the common mass action kinetics, written in brackets [10]. For convenience of notation, the matrices $\mathbf{N}^{\pm}$, whose elements $n_{ij}^{\pm}$ express the absolute values of the positive or negative stoichiometric coefficients, respectively, were introduced. All other kinetic equations presented in this section constitute special cases of this generalized form of the mass action rate law.

Feedback inhibition loops can be included with an appropriate choice of function F_j. Here we propose to adopt one of the following approaches with $K_j^{\mathrm{I}} \geqslant 0$:

$$F_j(\mathbf{S},\mathbf{p}) = \frac{1}{1+K_j^{\mathrm{I}}\cdot[\mathrm{I}]} \tag{3}$$

$$F_j(\mathbf{S},\mathbf{p}) = \exp(-K_j^{\mathrm{I}}\cdot[\mathrm{I}]). \tag{4}$$

Equation (3) can be derived as follows: An inhibitor lowers the concentration of free enzymes. Let $\eta_{\mathrm{E}}: \mathbb{R}\times\mathbb{R}_+ \to \mathbb{R}_+ \cup \{0\}$ be a function describing the efficiency factor of enzyme E for any reaction system

$$\mathrm{S}_1 + \mathrm{S}_2 \overset{\mathrm{E}}{\rightleftharpoons} \mathrm{P} \tag{5}$$

$$\mathrm{E} + \mathrm{I} \xrightleftharpoons{\text{spontaneous}} \mathrm{EI}, \tag{6}$$

where S_1 and S_2 form some product P inhibited by I.

In fact, instead of a bi-molecular reaction, we have a tri-molecular reaction due to the enzyme being involved. The mass action rate law for this reaction reads:

$$v = k_+[\mathrm{S}_1][\mathrm{S}_2][\mathrm{E}] - k_-[\mathrm{P}][\mathrm{E}]. \tag{7}$$

Assume the efficiency of enzyme E to be reduced by inhibitor I. The enzyme can either react with the two substrates or with the inhibitor molecule. Thus, the inhibitor reduces the concentration of the free enzyme $[\mathrm{E}_0]$:

$$\eta_{\mathrm{E}} = \frac{[\mathrm{E}_0]-[\mathrm{EI}]}{[\mathrm{E}_0]}. \tag{8}$$

E_0, EI and E follow a conservation law: $[\mathrm{E}_0] = [\mathrm{E}] + [\mathrm{EI}]$, where [E] is the enzyme concentration that is currently available to catalyze the reaction (5). We neglect the intermediary enzyme-substrate complex to simplify the notation. The equilibrium constant of reaction (6) is defined as

$$K^{\mathrm{I}} = \frac{[\mathrm{EI}]}{[\mathrm{E}]\cdot[\mathrm{I}]}. \tag{9}$$

Inserting Equation (9) into (8) and performing some conversion yields

$$\eta_{\mathrm{E}} = \frac{1}{1+K^{\mathrm{I}}\cdot[\mathrm{I}]}. \tag{10}$$

For any inhibition reaction two conditions must be guaranteed:

$$\eta_{\mathrm{E}}(t,0) = 1 \tag{11}$$

$$\lim_{[\mathrm{I}]\to\infty} \eta_{\mathrm{E}}(t,[\mathrm{I}]) = 0. \tag{12}$$

It can be shown easily that this function η_{E} evinces the desired properties needed to be a valid description of an inhibition reaction:

$$\begin{aligned}
\eta_{\mathrm{E}}(t,0) &= \frac{1}{1+K^{\mathrm{I}}\cdot 0} && =1 && \text{if} && [\mathrm{I}]=0\\
\eta_{\mathrm{E}}(t,[\mathrm{I}]) &= \frac{1}{1+0\cdot[\mathrm{I}]} && =1 && \text{if} && K^{\mathrm{I}}=0\\
\lim_{[\mathrm{I}]\to\infty} \eta_{\mathrm{E}}(t,[\mathrm{I}]) &= \lim_{[\mathrm{I}]\to\infty} \frac{1}{1+K^{\mathrm{I}}\cdot[\mathrm{I}]} && =0 && \text{if} && K^{\mathrm{I}}\neq 0.
\end{aligned}$$

For $K^{\mathrm{I}} = 0$ the inhibitor does not have any effect. Setting $F_j(\mathbf{S},\mathbf{p}) = \eta_{\mathrm{E}}$ and inserting it into Equation (2) yields the desired model.

The function η_{E} has been derived for bi-molecular reactions but can be scaled and applied to any number of inhibitors and reacting species as well. The general equation reads:

$$F_j(\mathbf{S},\mathbf{p}) = \prod_m \eta_{\mathrm{E}m}(t,S_m(t))^{w^-_{jm}}. \tag{13}$$

The elements of matrix $\mathbf{W}^-$ express the connectivity of modulators and reactions within the network in accordance with convenience kinetics.

Equation (4) has been derived intuitively, driven by the assumption that the exponent function constitutes an important growth and shrinkage function in biology. It is important to note that Equation (4) also satisfies both conditions of a valid inhibition function (Equations 11 and 12).

1.2 Michaelis-Menten Equation

The Michaelis-Menten equation can be applied to reactions in which one enzyme catalyzes the conversion of one substrate molecule into certain products, and it is sometimes called Henri-Michaelis-Menten equation [15, p. 30]. The general mechanism of this reaction is shown in Figure 1. Equation (14) gives the general equation as a special case of the generalized mass action kinetics for bi-molecular enzyme reactions of S and E forming product P and the catalyst E inhibited by I.

$$v_j = \frac{\frac{v^{\mathrm{m}}_{+}}{K^{\mathrm{M}}_{\mathrm{S}}}[\mathrm{S}] - \frac{v^{\mathrm{m}}_{-}}{K^{\mathrm{M}}_{\mathrm{P}}}[\mathrm{P}]}{1+\frac{[\mathrm{I}]}{K^{\mathrm{I}a}} + \left(\frac{[\mathrm{S}]}{K^{\mathrm{M}}_{\mathrm{S}}} + \frac{[\mathrm{P}]}{K^{\mathrm{M}}_{\mathrm{P}}}\right)\left(1+\frac{[\mathrm{I}]}{K^{\mathrm{I}b}}\right)} \tag{14}$$

Three limits upon the inhibition constants $K^{\mathrm{I}a|b}$ are often of particular interest [10]:

- competitive (for $0 < K^{\mathrm{I}a} < \infty$, $K^{\mathrm{I}b} \to \infty$)
- noncompetitive (for $0 < K^{\mathrm{I}a} = K^{\mathrm{I}b} < \infty$) and

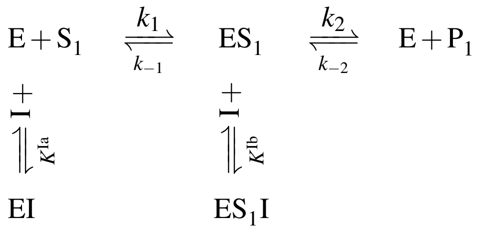

Figure 1: General Michaelis-Menten mechanism including inhibition

- uncompetitive inhibition (for $K^{Ia} \to \infty$, $0 < K^{Ib} < \infty$).

If the exact mechanism or the state at which the inhibitor binds to the enzyme is known, these constants may vanish. One purpose of this study is, however, to let an optimization procedure "decide", which kind of inhibition is the most appropriate one given *in vivo* measurements.

1.3 Convenience Rate Law and Thermodynamics

Recently, the convenience rate law was suggested by Liebermeister *et al.* [13] as a standard equation for any enzyme reaction where the exact mechanism is unknown or as an approximation of the real kinetics. The equation is derived from the random order ternary-complex reaction mechanism. It was shown that this rate law is able to describe the velocity of any reaction mechanism in a reasonable way [13] and hence constitutes a semi-mechanistic equation. The general equation of the convenience kinetics for reaction j reads:

$$v_j = [\mathrm{E}_j]\prod_m h_{\mathrm{A}}(S_m, K^{\mathrm{A}}_{jm})^{w^+_{jm}} h_{\mathrm{I}}(S_m, K^{\mathrm{I}}_{jm})^{w^-_{jm}} \cdot \frac{k^{\mathrm{cat}}_{+j}\prod_i \left(\frac{S_i}{K^{\mathrm{M}}_{ji}}\right)^{n^-_{ij}} - k^{\mathrm{cat}}_{-j}\prod_i \left(\frac{S_i}{K^{\mathrm{M}}_{ji}}\right)^{n^+_{ij}}}{\prod_i \sum_{m=0}^{n^-_{ij}} \left(\frac{S_i}{K^{\mathrm{M}}_{ji}}\right)^m + \prod_i \sum_{m=0}^{n^+_{ij}} \left(\frac{S_i}{K^{\mathrm{M}}_{ji}}\right)^m - 1} \tag{15}$$

with h_{A} and h_{I} being functions for activation and inhibition, respectively, $k^{\mathrm{cat}}_{\pm j}$ the turnover rates and K^{M}_{ji} being a constant analogous to the Michaelis constant K^{M} [13]. The modulation matrices $\mathbf{W}^{\pm}$ are defined in a similar way as the stoichiometric matrix and contain positive entries for the connectivity of the inhibitor or activator metabolites. Function h_{A} can be modeled in two alternative ways:

$$h_{\mathrm{A}}([\mathrm{S}_m], k^{\mathrm{A}}_{jm}) = \frac{[\mathrm{S}_m]}{k^{\mathrm{A}}_{jm} + [\mathrm{S}_m]} \tag{16}$$

$$h_{\mathrm{A}}([\mathrm{S}_m], k^{\mathrm{A}}_{jm}) = 1 + \frac{[\mathrm{S}_m]}{k^{\mathrm{A}}_{jm}}, \tag{17}$$

and for inhibition

$$h_{\mathrm{I}}(S_i, K^{\mathrm{I}}) = \frac{K^{\mathrm{I}}}{K^{\mathrm{I}} + S_i} = \frac{1}{1 + \frac{S_i}{K^{\mathrm{I}}}} = \frac{1}{1 + K^{\mathrm{I}\prime} S_i} = \eta_{\mathrm{E}} \tag{18}$$

was suggested [13]. This approach equals our inhibition in Equation (3) apart from the reciprocal constant. Equation (15) is also a special case of the generalized mass action kinetics and can be applied to any enzyme-catalyzed reaction. However, if the stoichiometric matrix **N** of the reaction system contains linearly dependent columns, i. e., **N** does not have full column rank, then at least one reaction is thermodynamically dependent on another. In this case, choosing the parameters of the equation while ignoring this dependency may fit given measurement data well but will violate the thermodynamic constraints of the system. Hence, Liebermeister *et al.* derived a second form of convenience kinetics:

$$v_j(\mathbf{S},\mathbf{p}) = [\mathrm{E}_j]\prod_m h_{\mathrm{A}}(S_m, K^{\mathrm{A}}_{jm})^{w^+_{jm}} h_{\mathrm{I}}(S_m, K^{\mathrm{I}}_{jm})^{w^-_{jm}}$$

$$\cdot k^{\mathrm{V}}_j \cdot [\mathrm{E}_j] \cdot \frac{\prod_i \left(\frac{[\mathrm{S}_i]}{K^{\mathrm{M}}_{ji}}\right)^{n^-_{ij}} \left(k^{\mathrm{G}}_i k^{\mathrm{M}}_{ji}\right)^{-\frac{n_{ij}}{2}} - \prod_i \left(\frac{[\mathrm{S}_i]}{K^{\mathrm{M}}_{ji}}\right)^{n^+_{ij}} \left(k^{\mathrm{G}}_i k^{\mathrm{M}}_{ji}\right)^{\frac{n_{ij}}{2}}}{\prod_i \sum_{m=0}^{n^-_{ij}} \left(\frac{[\mathrm{S}_i]}{K^{\mathrm{M}}_{ji}}\right)^m + \prod_i \sum_{m=0}^{n^+_{ij}} \left(\frac{[\mathrm{S}_i]}{K^{\mathrm{M}}_{ji}}\right)^m - 1}. \quad (19)$$

In this equation, the parameters $k^{\mathrm{cat}}_{\pm j}$ are replaced by $\prod_i \left(k^{\mathrm{G}}_i k^{\mathrm{M}}_{ji}\right)^{\mp\frac{n_{ij}}{2}}$ and the whole fraction is multiplied by the additional parameter k^{V}_j. This ensures that all newly introduced parameters are thermodynamically independent. Note that every k^{G}_i stands for molecule i regardless of the respective reaction, whereas every k^{V}_j is a parameter for reaction j and does not depend on any molecule. The Michaelis-analog parameter k^{M}_{ji} depends on both reaction j and molecule i and thus links both parameters together. For a complete derivation see the original paper of Liebermeister *et al.* [13].

1.4 Stochastic Langevin Equation

The use of ordinary differential equations to describe systems of chemical reactions implies that the underlying process is both continuous and deterministic. When taking the physical basis of chemical reactions into account, it is revealed that the evolution over time of a chemically reacting system is actually not a continuous process, because molecular population levels can only change by integral amounts. However, it is not a deterministic process, either. From the perspective of classical statistical mechanics, the precise knowledge of all particle positions and velocities is required to predict the temporal behavior of the system, which is, in principle, impossible to observe [7]. In many cases, of course, the application of the ordinary differential equation approach to chemical kinetics is justified, especially if the number of molecules per species is very large. In some cases, however, the inability of the deterministic approach to describe fluctuations in the molecular population levels can induce misleading results, especially if molecular concentrations are very low or the system operates close to a point of instability.

In the stochastic equation, the concentration variables S_i are replaced by the random variables $X_i(t) \equiv$ number of S_i molecules in the system at time t, $i = 1,\ldots,N$. These numbers are defined relative to an enclosed reaction volume V. These N species interact through M specified reaction channels R_j, $j = 1,\ldots,M$. Each reaction is characterized by a stochastic rate constant c_j, which describes for an infinitesimal time interval $\mathrm{d}t$ the probability that a particular combination of R_j

molecules will react accordingly in the next time interval dt [7]. This constant depends only on the physical properties of the reacting molecules.

The physically justified stochastic description of a system of chemically reacting species is given by the chemical master equation [16]:

$$\frac{\partial}{\partial t}P(X_1,\ldots,X_N;t) = \sum_{j=1}^{M} B_j - a_j P(X_1,\ldots,X_N;t). \tag{20}$$

This equation describes the temporal change of the grand probability function $P(X_1,\ldots,X_N;t) \equiv$ probability that there will be X_1 molecules of species $S_1,\ldots,$ and X_N molecules of species S_N in V at time t. Here the propensity a_j is defined as: $a_j\mathrm{d}t = c_j h_j \mathrm{d}t \equiv$ probability that an R_j reaction will occur in V in $(t, t+\mathrm{d}t)$, given that the system is in state $(X_1,\ldots,X_N)$ at time t. The function h_j gives the number of distinct R_j molecular reactant combinations available in the state $(X_1,\ldots,X_N)$, $j = 1,\ldots,M$. The quantity $B_j\mathrm{d}t$ is the probability that the system will undergo reaction R_j in the time interval $(t, t+\mathrm{d}t)$ to arrive at state $(X_1,\ldots,X_N)$.

For simulation studies, the master equation itself is of limited use, since even its numerical solution is difficult. Analytical solutions exist only for very few problems. Several strategies for the simulation of the underlying Markov process have been proposed [17]. In the case of large systems with high metabolite concentrations, these simulation strategies are highly computationally intensive and therefore unsuited for large-scale parameter optimization. However, for macroscopic systems it is possible to approximate the evolution in time of the stochastic state variables directly using the chemical Langevin equation [6, 7]:

$$\frac{\mathrm{d}X_i(t)}{\mathrm{d}t} = \sum_{j=1}^{M} n_{ij} a_j(\mathbf{X}(t)) + \sum_{j=1}^{M} \sqrt{a_j(\mathbf{X}(t))}\Gamma_j(t), \;\; i = 1,\ldots,N. \tag{21}$$

Here n_{ij} represents the stoichiometric coefficient of the ith metabolite in the jth reaction and $\Gamma_j(t)$ is temporally uncorrelated, statistically independent Gaussian white noise. In order for this approximation to hold, two requirements must be fulfilled:

1. There must exist a domain of macroscopically infinitesimal time intervals such that during any time interval dt all propensity functions remain approximately constant.

2. Each reaction channel is required to fire many more times than once. Large molecular populations will normally be an acceptable condition for the assumption to hold [6].

For easier numerical treatment, Equation (21) can be rewritten using the Wiener process [18]:

$$\mathrm{d}x_i(t) = \sum_{j=1}^{M} n_{ij} a_j(\mathbf{x}(t)) + \sum_{j=1}^{M} n_{ij}\sqrt{a_j(\mathbf{x}(t))}\mathrm{d}W_j, \; i = 1,\ldots,N. \tag{22}$$

Here the discrete variables $(X_1,\ldots,X_N)$ are replaced by the continuous molecule concentrations $(x_1,\ldots,x_N)$, since in the case of sufficiently high molecule concentrations both descriptions are considered equivalent. In order to numerically integrate the Langevin equation with standard

ordinary differential equation solvers, the equation can be split into a stochastic and a deterministic term. The deterministic term and the deterministic part of the stochastic term can be treated like ordinary differential equations as suggested by Bentele *et al.* [19]:

$$\Delta\hat{x}_i(t) = \sum_{j=1}^{M} n_{ij} a_j(\mathbf{x}_t)\Delta t \tag{23}$$

$$\Delta\tilde{x}_i(t) = \sum_{j=1}^{M} n_{ij} \sqrt{a_j(\mathbf{x}(t))\Delta t}. \tag{24}$$

The latter term is then multiplied by a normal random variable $n_i = \mathscr{N}$ in analogy with the finite Wiener increments used in the Euler-Maruyama method [18].

After each time-step, both terms are added to give the full state variable change:

$$\Delta x_i(t) = \Delta\hat{x}_i(t) + \Delta\tilde{x}_i(t) \cdot n_i \tag{25}$$

Using this relaxed Wiener process, knowledge of the step size Δt is not necessary ahead of time, thereby allowing adaptive step size control of a solver for ordinary differential equations.

2 Application to the Valine and Leucine Biosynthesis in *C. glutamicum*

Figure 2 depicts the valine and leucine biosynthesis in *Corynebacterium glutamicum* according to the METACYC [3] and KEGG [1, 2] databases. Table 1 gives an overview of all reactions within this network. Our consideration of the pathway starts with pyruvate (Pyr), which is subsequently consumed to form 2-ketoisovalerate (KIV) in three reaction steps. At this point the system contains a bifurcation: There are two different ways to form valine and one to convert KIV to 2-isopropylmalate (2IPM). The latter is the starting substance for leucine production in four following reaction steps. Both valine and leucine can be used for biomass production or can be transported out of the cell if not needed. Here we only consider the industrially interesting transport because it cannot be distinguished in the two processes. In four feedback loops valine and leucine downregulate their own production rate. The transport of leucine and valine across the cell wall is actually performed by the same enzyme, so both substrates compete with each other. However, for modeling purposes two distinct reactions are necessary in which the competition is included as inhibition.

Some reactions are lumped together (Table 1) as suggested by Magnus *et al.* [20]. Since the reaction $2\,\mathrm{IPM} \rightleftharpoons 3\,\mathrm{IPM}$ is fast, it is assumed to be in equilibrium. This and the two following reactions $3\,\mathrm{IPM} + \mathrm{NAD}^+ \longrightarrow 2\,\mathrm{I_3OS} + \mathrm{NADH_2}$ as well as (2S)-2-isopropyl-3-oxo-succinate ($2\,\mathrm{I_3OS}$) $\longrightarrow$ 2-ketoisocaproate (KIC) $+CO_2$, which only depend on the concentration of 2 IPM, are lumped together introducing the symbol IPM for both derivatives. [20]. The KEGG database [2, 1] mentions two additional reaction steps not included in METACYC [3]: Pyr turns first into 2-hydroxyethyl-thio-dipyrophosphate before forming (S)-2-acetolactate (AcLac) which then turns into 3-hydroxy-3-methyl-2-oxobutanoate before it forms (R)-2,3-dihydroxy-3-metylbutanoate (DHIV).

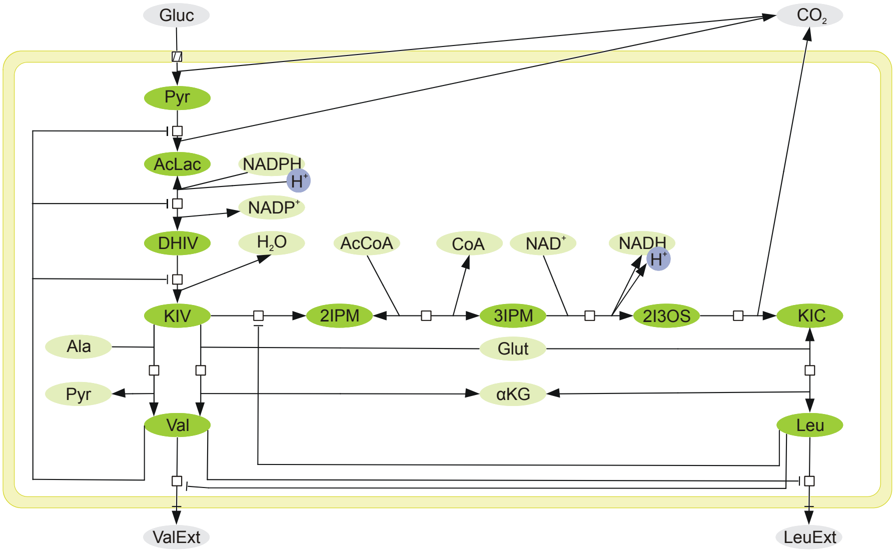

Figure 2: Process diagram of the valine and leucine synthesis in *C. glutamicum*
Note that enzyme molecules are not included in this process diagram for the sake of a clear arrangement of the participating species.

№	Reaction	Enzyme	Inhibitor
R_1	$2\,\mathrm{Pyr} \longrightarrow \mathrm{AcLac} + \mathrm{CO_2}$	AHAS	Val
R_2	$\mathrm{AcLac} + \mathrm{NADPH_2} \rightleftharpoons \mathrm{DHIV} + \mathrm{NADP^+}$	AHAIR	Val
R_3	$\mathrm{DHIV} \longrightarrow \mathrm{KIV} + \mathrm{H_2O}$	DHAD	Val
R_4	$\mathrm{KIV} + \mathrm{Glut} \longrightarrow \mathrm{Val} + \alpha\mathrm{KG}$	$\mathrm{BCAAT_{ValB}}$	
R_5	$\mathrm{KIV} + \mathrm{Ala} \longrightarrow \mathrm{Val} + \mathrm{Pyr}$	$\mathrm{BCAAT_{ValC}}$	
R_6	$\mathrm{Val} \longrightarrow \mathrm{Val_{ext}}$	$\mathrm{Trans_{Val}}$	Leu
R_7	$\mathrm{KIV} + \mathrm{AcCoA} \longrightarrow \mathrm{IPM} + \mathrm{CoA}$	IPMS	Leu
R_8	$\mathrm{IPM} + \mathrm{NAD^+} \longrightarrow \mathrm{KIC} + \mathrm{NADH_2} + \mathrm{CO_2}$	IPMDH	
R_9	$\mathrm{KIC} + \mathrm{Glut} \rightleftharpoons \mathrm{Leu} + \alpha\mathrm{KG}$	$\mathrm{BCAAT_{LeuB}}$	
R_{10}	$\mathrm{Leu} \longrightarrow \mathrm{Leu_{ext}}$	$\mathrm{Trans_{Leu}}$	Val

Table 1: The reaction system in more detail
This table gives an overview of all reactions in the system.

When applying Equation 1 to the reactions listed in Table 1 the resulting seven-dimensional differential equation system reads:

$$\frac{\mathrm{d}}{\mathrm{d}t}[\mathrm{AcLac}] = v_1 - v_2 \tag{26}$$
$$\frac{\mathrm{d}}{\mathrm{d}t}[\mathrm{DHIV}] = v_2 - v_3 \tag{27}$$
$$\frac{\mathrm{d}}{\mathrm{d}t}[\mathrm{KIV}] = v_3 - v_4 - v_5 - v_7 \tag{28}$$
$$\frac{\mathrm{d}}{\mathrm{d}t}[\mathrm{IPM}] = v_7 - v_8 \tag{29}$$
$$\frac{\mathrm{d}}{\mathrm{d}t}[\mathrm{Val}] = v_4 + v_5 - v_6 \tag{30}$$
$$\frac{\mathrm{d}}{\mathrm{d}t}[\mathrm{KIC}] = v_8 - v_9 \tag{31}$$
$$\frac{\mathrm{d}}{\mathrm{d}t}[\mathrm{Leu}] = v_9 - v_{10}. \tag{32}$$

The remainder of this section explains how external metabolites could be included in this equation system and then presents seven alternative formulations for the reaction velocities based on the four approaches that were introduced in the last section. The seven-dimensional differential equation system shown above does not vary with alternative choices of approximative rate laws.

2.1 Modeling External Metabolites Using Approximation Splines

Six metabolites are consumed or formed in this network but take part in several reactions not considered here. These are called "external" metabolites because the temporal changes of their concentration cannot be computed in terms of this network. These six metabolites, α-ketoglutarate (αKG), alanine (Ala), glutamate (Glut), pyruvate (Pyr), $NADP^+$ and NAD^+ are therefore included using splines. Instead of using exact splines that connect all measurements, we use cubic approximation splines. This kind of spline is defined by an at-least-twice differentiable cubic polynomial with four coefficients between each pair of two measurements. For the given measurements $(t_n, x_{t_n i})$, for all metabolites i, $n = 1, \ldots, N$ the spline coefficients are chosen as the solution of the minimization problem

$$\min_{f_i \in \mathfrak{C}^2(t_1, t_N)} \int_{t_1}^{t_N} (f_i''(t))^2 \mathrm{d}t \tag{33}$$

satisfying the constraint

$$\sum_{i=1}^{N} \left(\frac{f(t_i) - x_{ti}}{\omega_i} \right)^2 \leqslant \lambda \tag{34}$$

where N is the total number of time points, ω is a vector of weights and the parameter λ specifies the degree of smoothness. To weight all measurements equally, all ω_i are set to 1. Due to the different ranges of the concentrations of the six metabolites, it is not possible to find one appropriate degree of smoothness λ that leads to equally smooth curves. Hence, we transform all concentrations into the range $[0, 1]$, set $\lambda = 1$, compute the spline coefficients and re-transform them back into the original range of the specific metabolite. The result is shown in Figure 3.

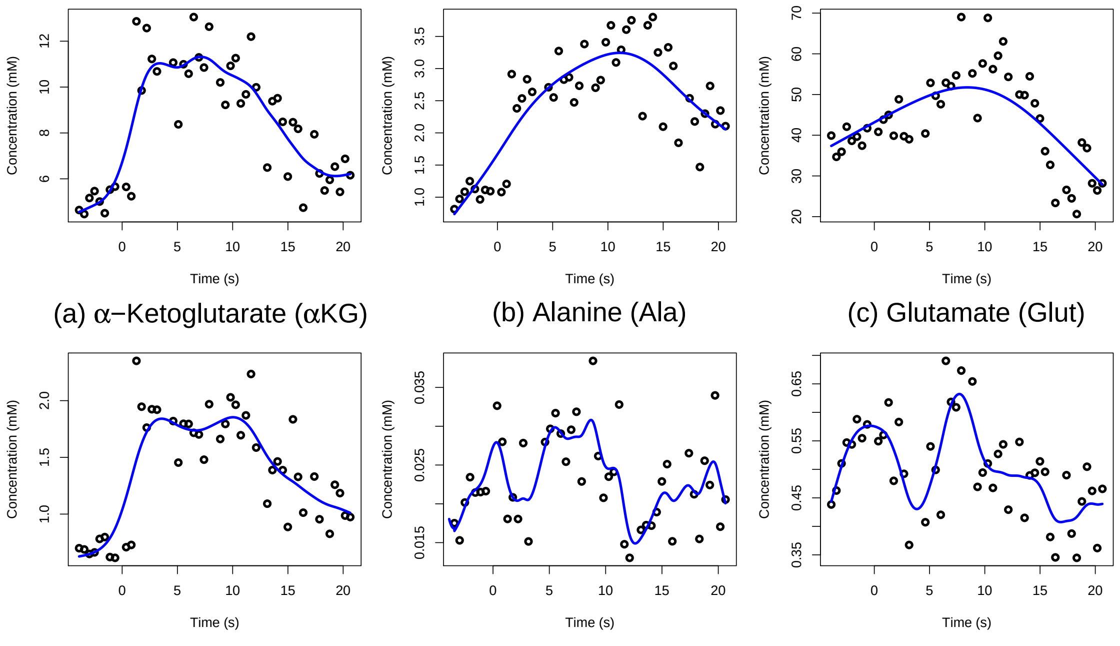

(a) α−Ketoglutarate (αKG) (b) Alanine (Ala) (c) Glutamate (Glut)

(d) Pyruvate (Pyr) (e) $NADP^+$ (f) NAD^+

Figure 3: Representing external metabolites using approximation splines

Six of the 13 measured metabolites are considered external because these metabolites are an input for the model but occur in many other reactions which are not part of this network. To include their dynamic behavior into the model, they are approximated with cubic splines. Splines smooth the fluctuating measurements but do not depend on any biologically relevant model since their coefficients are computed individually for each chemical species.

2.2 Generalized Mass Action Rate Law

2.2.1 Reversible Reactions (GMAKr)

Applying Equation (2), combined with Equation (3), to reaction system R_1 through R_{10} (Table 1) leads to an ordinary differential equation system with 24 parameters $k_{\pm j}$, K^{I}_j:

$$v_1 = \frac{k_{+1}[\mathrm{Pyr}]^2 - k_{-1}[\mathrm{AcLac}]}{1 + K^{\mathrm{I}}_1[\mathrm{Val}]} \tag{35}$$

$$v_2 = \frac{k_{+2}[\mathrm{AcLac}][\mathrm{NADPH_2}]}{1 + K^{\mathrm{I}}_2[\mathrm{Val}]} - \frac{k_{-2}[\mathrm{DHIV}][\mathrm{NADP^+}]}{1 + K^{\mathrm{I}}_2[\mathrm{Val}]} \tag{36}$$

$$v_3 = \frac{k_{+3}[\mathrm{DHIV}] - k_{-3}[\mathrm{KIV}]}{1 + K^{\mathrm{I}}_3[\mathrm{Val}]} \tag{37}$$

$$v_4 = k_{+4}[\mathrm{KIV}][\mathrm{Glut}] - k_{-4}[\mathrm{Val}][\alpha\mathrm{KG}] \tag{38}$$

$$v_5 = k_{+5}[\mathrm{KIV}][\mathrm{Ala}] - k_{-5}[\mathrm{Val}][\mathrm{Pyr}] \tag{39}$$

$$v_6 = \frac{k_{+6}[\mathrm{Val}]}{1 + K_4^{\mathrm{I}}[\mathrm{Leu}]} \tag{40}$$

$$v_7 = \frac{k_{+7}[\mathrm{KIV}][\mathrm{AcCoA}] - k_{-7}[\mathrm{IPM}][\mathrm{CoA}]}{1 + K_5^{\mathrm{I}}[\mathrm{Leu}]} \tag{41}$$

$$v_8 = k_{+8}[\mathrm{IPM}][\mathrm{NAD}^+] - k_{-8}[\mathrm{KIC}][\mathrm{NADH}_2] \tag{42}$$

$$v_9 = k_{+9}[\mathrm{KIC}][\mathrm{Glut}] - k_{-9}[\mathrm{Leu}][\alpha\mathrm{KG}] \tag{43}$$

$$v_{10} = \frac{k_{+10}[\mathrm{Leu}]}{1 + K_6^{\mathrm{I}}[\mathrm{Val}]} \tag{44}$$

2.2.2 Irreversible Reactions with exp Inhibition (GMAKi)

By setting all product concentrations, apart from R_2 and R_9, to zero and applying Equation (4) to Equation (2), we obtain the irreversible version of this equation system with 18 parameters $k_{\pm j}$, K_j^{I}:

$$v_1 = k_{+1}[\mathrm{Pyr}]^2 \exp(-K_1^{\mathrm{I}}[\mathrm{Val}]) \tag{45}$$

$$v_2 = \exp(-K_2^{\mathrm{I}}[\mathrm{Val}]) \cdot \big(k_{+2}[\mathrm{AcLac}][\mathrm{NADPH}_2] - k_{-2}[\mathrm{DHIV}][\mathrm{NADP}^+]\big) \tag{46}$$

$$v_3 = k_{+3}[\mathrm{DHIV}] \exp(-K_3^{\mathrm{I}}[\mathrm{Val}]) \tag{47}$$

$$v_4 = k_{+4}[\mathrm{Glut}][\mathrm{KIV}] \tag{48}$$

$$v_5 = k_{+5}[\mathrm{Ala}][\mathrm{KIV}] \tag{49}$$

$$v_6 = k_{+6}[\mathrm{Val}] \exp(-K_4^{\mathrm{I}}[\mathrm{Leu}]) \tag{50}$$

$$v_7 = k_{+7}[\mathrm{KIV}] \exp(-K_5^{\mathrm{I}}[\mathrm{Leu}]) \tag{51}$$

$$v_8 = k_{+8}[\mathrm{NAD}^+][\mathrm{IPM}] \tag{52}$$

$$v_9 = k_{+9}[\mathrm{Glut}][\mathrm{KIC}] - k_{-9}[\alpha\mathrm{KG}][\mathrm{Leu}] \tag{53}$$

$$v_{10} = k_{+10}[\mathrm{Leu}] \exp(-K_6^{\mathrm{I}}[\mathrm{Val}].) \tag{54}$$

2.3 Michaelis-Menten Equations

Three reactions of the system (R_3, R_6 and R_{10}, Table 1) follow a bi-molecular Michaelis-Menten reaction mechanism. In the case of R_3 there might be a reverse reaction. Both of the remaining reactions are assumed to be irreversible because they describe the transport of valine and leucine out of the cell. Since we model the production of both metabolic products, there is no reason to have an uptake mechanism for these substances. We further assume that both v_{+6}^{m} and v_{+10}^{m} are allowed to be zero so there is no need to export valine or leucine if it is necessary for biomass formation. The other reactions in the equation system (35) through (57) are modeled using the GMAKr approach including inhibition mechanism (3) derived in Section 2.2.1. The complete GMMr model contains 31 parameters to be estimated. To avoid numerical problems, the inhibition constants in Michaelis-Menten kinetics are transformed into their reciprocals $K^{\mathrm{I}a|b\prime} = \frac{1}{K^{\mathrm{I}a|b}}$. This modification allows us to model any kind of inhibition. Three particularly important special cases were described in Section 2.3.1. For instance, by setting $K^{\mathrm{I}a|b\prime} = 0$ we obtain the same

effect as if $K^{\mathrm{I}a|b} \to \infty$ and avoid numerical problems. For the sake of simplicity we omit the prime symbol in the following equations.

2.3.1 Reversible Michaelis-Menten Model (GMMr)

Replacing v_3, v_6 and v_{10} in the GMAKr model with the following three equations yields the GMMr model:

$$v_3 = \frac{\frac{v^{\mathrm{m}}_{+3}}{K^{\mathrm{M}}_{[\mathrm{DHIV}]}}[\mathrm{DHIV}] - \frac{v^{\mathrm{m}}_{-3}}{K^{\mathrm{M}}_{[\mathrm{KIV}]}}[\mathrm{KIV}]}{1 + K^{\mathrm{I}a}_1[\mathrm{Val}] + \left(\frac{[\mathrm{DHIV}]}{K^{\mathrm{M}}_{[\mathrm{DHIV}]}} + \frac{[\mathrm{KIV}]}{K^{\mathrm{M}}_{[\mathrm{KIV}]}}\right)\left(1 + K^{\mathrm{I}b}_1[\mathrm{Val}]\right)} \tag{55}$$

$$v_6 = \frac{v^{\mathrm{m}}_{+6}[\mathrm{Val}]}{K^{\mathrm{M}}_{[\mathrm{Val}]} + [\mathrm{Val}] + \left(K^{\mathrm{M}}_{[\mathrm{Val}]}K^{\mathrm{I}a}_2 + K^{\mathrm{I}b}_2[\mathrm{Val}]\right)[\mathrm{Leu}]} \tag{56}$$

$$v_{10} = \frac{v^{\mathrm{m}}_{+10}[\mathrm{Leu}]}{K^{\mathrm{M}}_{[\mathrm{Leu}]} + [\mathrm{Leu}] + \left(K^{\mathrm{M}}_{[\mathrm{Leu}]}K^{\mathrm{I}a}_3 + K^{\mathrm{I}b}_3[\mathrm{Leu}]\right)[\mathrm{Val}]}. \tag{57}$$

2.3.2 Irreversible Michaelis-Menten Model (GMMi)

An irreversible alternative of the GMMr model can be established by setting all product concentrations to zero. The resulting system contains 24 parameters $K^{\mathrm{Ia|b}}_j$, $k_{\pm j}$, K^{M}_{ij}:

$$v_1 = \frac{k_{+1}[\mathrm{Pyr}]^2}{1 + K^{\mathrm{I}}_1[\mathrm{Val}]} \tag{58}$$

$$v_2 = \frac{k_{+2}[\mathrm{AcLac}][\mathrm{NADPH_2}]}{1 + K^{\mathrm{I}}_2[\mathrm{Val}]} - \frac{k_{-2}[\mathrm{DHIV}][\mathrm{NADP^+}]}{1 + K^{\mathrm{I}}_2[\mathrm{Val}]} \tag{59}$$

$$v_3 = \frac{\frac{v^{\mathrm{m}}_{+3}}{K^{\mathrm{M}}_{[\mathrm{DHIV}]}}[\mathrm{DHIV}]}{1 + K^{\mathrm{I}a}_1[\mathrm{Val}] + \frac{[\mathrm{DHIV}]}{K^{\mathrm{M}}_{[\mathrm{DHIV}]}}\left(1 + K^{\mathrm{I}b}_1[\mathrm{Val}]\right)} \tag{60}$$

$$v_4 = k_{+4}[\mathrm{KIV}][\mathrm{Glut}] \tag{61}$$

$$v_5 = k_{+5}[\mathrm{KIV}][\mathrm{Ala}] \tag{62}$$

$$v_6 = \frac{v^{\mathrm{m}}_{+6}[\mathrm{Val}]}{K^{\mathrm{M}}_{[\mathrm{Val}]} + [\mathrm{Val}] + \left(K^{\mathrm{M}}_{[\mathrm{Val}]}K^{\mathrm{I}a}_2 + K^{\mathrm{I}b}_2[\mathrm{Val}]\right)[\mathrm{Leu}]} \tag{63}$$

$$v_7 = \frac{k_{+7}[\mathrm{KIV}][\mathrm{AcCoA}]}{1 + K^{\mathrm{I}}_5[\mathrm{Leu}]} \tag{64}$$

$$v_8 = k_{+8}[\mathrm{IPM}][\mathrm{NAD^+}] \tag{65}$$

$$v_9 = k_{+9}[\mathrm{KIC}][\mathrm{Glut}] - k_{-9}[\mathrm{Leu}][\alpha\mathrm{KG}] \tag{66}$$

$$v_{10} = \frac{v^{\mathrm{m}}_{+10}[\mathrm{Leu}]}{K^{\mathrm{M}}_{[\mathrm{Leu}]} + [\mathrm{Leu}] + \left(K^{\mathrm{M}}_{[\mathrm{Leu}]}K^{\mathrm{I}a}_3 + K^{\mathrm{I}b}_3[\mathrm{Leu}]\right)[\mathrm{Val}]}. \tag{67}$$

2.4 Convenience Kinetics Model

Inhibition plays an important role in the valine and leucine biosynthesis of *C. glutamicum*. Therefore, Equation (18) is applied to include those effects.

2.4.1 Reversible Convenience Kinetics (CKMMr)

The stoichiometric matrix has full column rank. Hence, the parameters $k^{\mathrm{cat}}_{\pm j}$ can be estimated directly without violating thermodynamic constraints [13]. Therefore, the simple form of the convenience rate law, which contains a smaller number of parameters, is applied in this study. Applying Equation (15) to reaction system R_1 through R_{10} yields the equation system (68)-(74). The three reactions that follow the traditional bi-molecular Michaelis-Menten mechanism are modeled using Equation (14) and can be found in Section 2.3.1. The reactions R_6 and R_{10} are considered irreversible as described before. The product $[\mathrm{E}_j]k^{\mathrm{cat}}_{\pm j}$ is lumped into one parameter $V^{\mathrm{m}}_{\pm j}$ for all j assuming all enzyme concentrations to remain constant during the 25 s. No enzyme concentrations have been measured, so an optimizer cannot distinguish between the product of two parameters and that of one parameter, due to the infinite number of combinations leading to the same product. The whole system contains 59 parameters.

$$v_1 = \frac{\frac{k^{\mathrm{cat}}_{+1}\cdot[\mathrm{AHAS}]\cdot K^{\mathrm{I}}_1}{\left(K^{\mathrm{M}}_{[\mathrm{Pyr}]1}\right)^2}[\mathrm{Pyr}]^2 - \frac{k^{\mathrm{cat}}_{-1}\cdot[\mathrm{AHAS}]\cdot K^{\mathrm{I}}_1}{K^{\mathrm{M}}_{[\mathrm{AcLac}]1}}[\mathrm{AcLac}]}{\left(1+\frac{[\mathrm{Pyr}]}{K^{\mathrm{M}}_{[\mathrm{Pyr}]1}}+\left(\frac{[\mathrm{Pyr}]}{K^{\mathrm{M}}_{[\mathrm{Pyr}]1}}\right)^2+\frac{[\mathrm{AcLac}]}{K^{\mathrm{M}}_{[\mathrm{AcLac}]1}}\right)\left(K^{\mathrm{I}}_1+[\mathrm{Val}]\right)} \tag{68}$$

$$v_2 = \frac{\frac{k^{\mathrm{cat}}_{+2}\cdot[\mathrm{AHAIR}]\cdot K^{\mathrm{I}}_2}{K^{\mathrm{M}}_{[\mathrm{AcLac}]2}\cdot K^{\mathrm{M}}_{[\mathrm{NADPH_2}]1}}[\mathrm{AcLac}][\mathrm{NADPH_2}] - \frac{k^{\mathrm{cat}}_{-2}\cdot[\mathrm{AHAIR}]\cdot K^{\mathrm{I}}_2}{K^{\mathrm{M}}_{[\mathrm{DHIV}]1}\cdot K^{\mathrm{M}}_{[\mathrm{NADP^+}]1}}[\mathrm{DHIV}][\mathrm{NADP^+}]}{\left(1+\frac{[\mathrm{AcLac}]}{K^{\mathrm{M}}_{[\mathrm{AcLac}]2}}\right)\left(1+\frac{[\mathrm{NADPH_2}]}{K^{\mathrm{M}}_{[\mathrm{NADPH_2}]1}}\right)+\left(1+\frac{[\mathrm{DHIV}]}{K^{\mathrm{M}}_{[\mathrm{DHIV}]1}}\right)\left(1+\frac{[\mathrm{NADP^+}]}{K^{\mathrm{M}}_{[\mathrm{NADP^+}]1}}\right)-1} \cdot \frac{1}{K^{\mathrm{I}}_2+[\mathrm{Val}]} \tag{69}$$

$$v_4 = \frac{\frac{k^{\mathrm{cat}}_{+4}\cdot[\mathrm{BCAAT_{ValB}}]}{K^{\mathrm{M}}_{[\mathrm{KIV}]1}\cdot K^{\mathrm{M}}_{[\mathrm{Glut}]1}}[\mathrm{KIV}][\mathrm{Glut}] - \frac{k^{\mathrm{cat}}_{-4}\cdot[\mathrm{BCAAT_{ValB}}]}{K^{\mathrm{M}}_{[\mathrm{Val}]1}\cdot K^{\mathrm{M}}_{[\alpha\mathrm{KG}]1}}[\mathrm{Val}][\alpha\mathrm{KG}]}{1+\frac{[\mathrm{KIV}]}{K^{\mathrm{M}}_{[\mathrm{KIV}]1}}+\frac{[\mathrm{Glut}]}{K^{\mathrm{M}}_{[\mathrm{Glut}]1}}+\frac{[\mathrm{KIV}][\mathrm{Glut}]}{K^{\mathrm{M}}_{[\mathrm{KIV}]1}\cdot K^{\mathrm{M}}_{[\mathrm{Glut}]1}}+\frac{[\mathrm{Val}]}{K^{\mathrm{M}}_{[\mathrm{Val}]1}}+\frac{[\alpha\mathrm{KG}]}{K^{\mathrm{M}}_{[\alpha\mathrm{KG}]1}}+\frac{[\mathrm{Val}][\alpha\mathrm{KG}]}{K^{\mathrm{M}}_{[\mathrm{Val}]1}\cdot K^{\mathrm{M}}_{[\alpha\mathrm{KG}]1}}} \tag{70}$$

$$v_5 = \frac{\frac{k^{\mathrm{cat}}_{+5}\cdot[\mathrm{BCAAT_{ValC}}]}{K^{\mathrm{M}}_{[\mathrm{KIV}]2}\cdot K^{\mathrm{M}}_{[\mathrm{Ala}]1}}[\mathrm{KIV}][\mathrm{Ala}] - \frac{k^{\mathrm{cat}}_{-5}\cdot[\mathrm{BCAAT_{ValC}}]}{K^{\mathrm{M}}_{[\mathrm{Val}]2}\cdot K^{\mathrm{M}}_{[\mathrm{Pyr}]2}}[\mathrm{Val}][\mathrm{Pyr}]}{1+\frac{[\mathrm{KIV}]}{K^{\mathrm{M}}_{[\mathrm{KIV}]2}}+\frac{[\mathrm{Ala}]}{K^{\mathrm{M}}_{[\mathrm{Ala}]1}}+\frac{[\mathrm{KIV}][\mathrm{Ala}]}{K^{\mathrm{M}}_{[\mathrm{KIV}]2}\cdot K^{\mathrm{M}}_{[\mathrm{Ala}]1}}+\frac{[\mathrm{Val}]}{K^{\mathrm{M}}_{[\mathrm{Val}]2}}+\frac{[\mathrm{Pyr}]}{K^{\mathrm{M}}_{[\mathrm{Pyr}]2}}+\frac{[\mathrm{Val}][\mathrm{Pyr}]}{K^{\mathrm{M}}_{[\mathrm{Val}]2}\cdot K^{\mathrm{M}}_{[\mathrm{Pyr}]2}}} \tag{71}$$

$$v_7 = \frac{\frac{k^{\mathrm{cat}}_{+7}\cdot[\mathrm{IPMS}]\cdot K^{\mathrm{I}}_3}{K^{\mathrm{M}}_{[\mathrm{KIV}]3}\cdot K^{\mathrm{M}}_{[\mathrm{AcCoA}]1}}[\mathrm{KIV}][\mathrm{AcCoA}] - \frac{k^{\mathrm{cat}}_{-7}\cdot[\mathrm{IPMS}]\cdot K^{\mathrm{I}}_3}{K^{\mathrm{M}}_{[\mathrm{IPM}]1}\cdot K^{\mathrm{M}}_{[\mathrm{CoA}]1}}[\mathrm{IPM}][\mathrm{CoA}]}{1+\frac{[\mathrm{KIV}]}{K^{\mathrm{M}}_{[\mathrm{KIV}]3}}+\frac{[\mathrm{AcCoA}]}{K^{\mathrm{M}}_{[\mathrm{AcCoA}]1}}+\frac{[\mathrm{KIV}][\mathrm{AcCoA}]}{K^{\mathrm{M}}_{[\mathrm{KIV}]3}\cdot K^{\mathrm{M}}_{[\mathrm{AcCoA}]1}}+\frac{[\mathrm{IPM}]}{K^{\mathrm{M}}_{[\mathrm{IPM}]1}}+\frac{[\mathrm{CoA}]}{K^{\mathrm{M}}_{[\mathrm{CoA}]1}}+\frac{[\mathrm{IPM}][\mathrm{CoA}]}{K^{\mathrm{M}}_{[\mathrm{IPM}]1}K^{\mathrm{M}}_{[\mathrm{CoA}]1}}} \cdot \frac{1}{\left(K^{\mathrm{I}}_3+[\mathrm{Leu}]\right)} \tag{72}$$

$$v_8 = \frac{\frac{k^{\text{cat}}_{+8}\cdot[\text{IPMDH}]}{K^{\text{M}}_{[\text{IPM}]2}\cdot K^{\text{M}}_{[\text{NAD}^+]1}}[\text{IPM}][\text{NAD}^+] - \frac{k^{\text{cat}}_{-8}\cdot[\text{IPMDH}]}{K^{\text{M}}_{[\text{KIC}]1}\cdot K^{\text{M}}_{[\text{NADH}_2]1}}[\text{KIC}][\text{NADH}_2]}{1+\frac{[\text{IPM}]}{K^{\text{M}}_{[\text{IPM}]2}}+\frac{[\text{NAD}^+]}{K^{\text{M}}_{[\text{NAD}^+]1}}+\frac{[\text{IPM}][\text{NAD}^+]}{K^{\text{M}}_{[\text{IPM}]2}\cdot K^{\text{M}}_{[\text{NAD}^+]1}}+\frac{[\text{KIC}]}{K^{\text{M}}_{[\text{KIC}]1}}+\frac{[\text{NADH}_2]}{K^{\text{M}}_{[\text{NADH}_2]1}}+\frac{[\text{KIC}][\text{NADH}_2]}{K^{\text{M}}_{[\text{NADH}_2]1}K^{\text{M}}_{[\text{KIC}]1}}} \tag{73}$$

$$v_9 = \frac{\frac{k^{\text{cat}}_{+9}\cdot[\text{BCAAT}_{\text{LeuB}}]}{K^{\text{M}}_{[\text{KIC}]2}\cdot K^{\text{M}}_{[\text{Glut}]2}}[\text{KIC}][\text{Glut}] - \frac{k^{\text{cat}}_{-9}\cdot[\text{BCAAT}_{\text{LeuB}}]}{K^{\text{M}}_{[\text{Leu}]1}\cdot K^{\text{M}}_{[\alpha\text{KG}]2}}[\text{Leu}][\alpha\text{KG}]}{1+\frac{[\text{KIC}]}{K^{\text{M}}_{[\text{KIC}]2}}+\frac{[\text{Glut}]}{K^{\text{M}}_{[\text{Glut}]2}}+\frac{[\text{KIC}][\text{Glut}]}{K^{\text{M}}_{[\text{KIC}]2}\cdot K^{\text{M}}_{[\text{Glut}]2}}+\frac{[\text{Leu}]}{K^{\text{M}}_{[\text{Leu}]1}}+\frac{[\alpha\text{KG}]}{K^{\text{M}}_{[\alpha\text{KG}]2}}+\frac{[\text{Leu}][\alpha\text{KG}]}{K^{\text{M}}_{[\text{Leu}]1}\cdot K^{\text{M}}_{[\alpha\text{KG}]2}}} \tag{74}$$

2.4.2 Irreversible Convenience Kinetics (CKMMi)

By setting all product concentrations, apart from R_2 and R_9, to zero, we obtain an irreversible version of this model containing 41 parameters:

$$v_1 = \frac{\frac{k^{\text{cat}}_{+1}\cdot[\text{AHAS}]\cdot K^{\text{I}}_1}{\left(K^{\text{M}}_{[\text{Pyr}]1}\right)^2}[\text{Pyr}]^2}{\left(1+\frac{[\text{Pyr}]}{K^{\text{M}}_{[\text{Pyr}]1}}+\left(\frac{[\text{Pyr}]}{K^{\text{M}}_{[\text{Pyr}]1}}\right)^2\right)\left(K^{\text{I}}_1+[\text{Val}]\right)} \tag{75}$$

$$v_4 = \frac{\frac{k^{\text{cat}}_{+4}\cdot[\text{BCAAT}_{\text{ValB}}]}{K^{\text{M}}_{[\text{KIV}]1}\cdot K^{\text{M}}_{[\text{Glut}]1}}[\text{KIV}][\text{Glut}]}{1+\frac{[\text{KIV}]}{K^{\text{M}}_{[\text{KIV}]1}}+\frac{[\text{Glut}]}{K^{\text{M}}_{[\text{Glut}]1}}+\frac{[\text{KIV}][\text{Glut}]}{K^{\text{M}}_{[\text{KIV}]1}\cdot K^{\text{M}}_{[\text{Glut}]1}}} \tag{76}$$

$$v_5 = \frac{\frac{k^{\text{cat}}_{+5}\cdot[\text{BCAAT}_{\text{ValC}}]}{K^{\text{M}}_{[\text{KIV}]2}\cdot K^{\text{M}}_{[\text{Ala}]1}}[\text{KIV}][\text{Ala}]}{1+\frac{[\text{KIV}]}{K^{\text{M}}_{[\text{KIV}]2}}+\frac{[\text{Ala}]}{K^{\text{M}}_{[\text{Ala}]1}}+\frac{[\text{KIV}][\text{Ala}]}{K^{\text{M}}_{[\text{KIV}]2}\cdot K^{\text{M}}_{[\text{Ala}]1}}} \tag{77}$$

$$v_7 = \frac{\frac{k^{\text{cat}}_{+7}\cdot[\text{IPMS}]\cdot K^{\text{I}}_3}{K^{\text{M}}_{[\text{KIV}]3}\cdot K^{\text{M}}_{[\text{AcCoA}]1}}[\text{KIV}][\text{AcCoA}]}{\left(1+\frac{[\text{KIV}]}{K^{\text{M}}_{[\text{KIV}]3}}+\frac{[\text{AcCoA}]}{K^{\text{M}}_{[\text{AcCoA}]1}}+\frac{[\text{KIV}][\text{AcCoA}]}{K^{\text{M}}_{[\text{KIV}]3}\cdot K^{\text{M}}_{[\text{AcCoA}]1}}\right)\left(K^{\text{I}}_3+[\text{Leu}]\right)} \tag{78}$$

$$v_8 = \frac{\frac{k^{\text{cat}}_{+8}\cdot[\text{IPMDH}]}{K^{\text{M}}_{[\text{IPM}]2}\cdot K^{\text{M}}_{[\text{NAD}^+]1}}[\text{IPM}][\text{NAD}^+]}{1+\frac{[\text{IPM}]}{K^{\text{M}}_{[\text{IPM}]2}}+\frac{[\text{NAD}^+]}{K^{\text{M}}_{[\text{NAD}^+]1}}+\frac{[\text{IPM}][\text{NAD}^+]}{K^{\text{M}}_{[\text{IPM}]2}\cdot K^{\text{M}}_{[\text{NAD}^+]1}}} \tag{79}$$

2.5 Stochastic Modeling based on the Langevin Equation (LANG)

It is expected that in the pathway under consideration neither the concentrations of participating molecules are very low nor the system operates close to a point of instability. To demonstrate the possibility of large-scale parameter optimization even for stochastic models and to model the effects of random fluctuations in the metabolite concentrations, we consider a stochastic description, based on the Langevin approach [6].

In the system under study a domain of macroscopically infinitesimal time intervals exists, as required by condition 1 in Section 1.4, page 9, and each reaction fires many more times than once due to the large molecular population (condition 2). The reaction propensities are calculated

according to Gillespie [7]. The propensities for the standard are essentially proportional to the product of the number of participating molecules. For reactions that involve inhibition, we assume that the propensity is inversely proportional to the inhibitor concentrations, similar to the inhibition term occurring in the GMAKr model. This leads to the following equation system for the stochastic simulation with 24 parameters:

$$v_1 = \frac{c_1[\mathrm{PYR}]^2}{2+2C_1[\mathrm{Val}]} + \sqrt{\frac{c_1}{2+2C_1\mathrm{Val}}}[\mathrm{PYR}]\,\Gamma_1(t) - \frac{c_{11}[\mathrm{AcLac}]}{1+C_1[\mathrm{Val}]} - \sqrt{\frac{c_{11}[\mathrm{AcLac}]}{1+C_1\mathrm{Val}}}\,\Gamma_{11}(t) \quad (80)$$

$$v_2 = \frac{c_2[\mathrm{AcLac}][\mathrm{NADPH_2}] - c_{12}[\mathrm{DHIV}][\mathrm{NADP^+}]}{1+C_2[\mathrm{Val}]} + \frac{\sqrt{c_2[\mathrm{AcLac}][\mathrm{NADPH_2}]}\,\Gamma_2(t) - \sqrt{c12[\mathrm{DHIV}][\mathrm{NADP^+}]}\,\Gamma_{12}(t))}{\sqrt{1+C_2[\mathrm{Val}]}} \quad (81)$$

$$v_3 = \frac{c_3[\mathrm{DHIV}] - c_{13}[\mathrm{KIV}]}{1+C_3[\mathrm{Val}]} + \frac{\sqrt{c_3[\mathrm{DHIV}]}\,\Gamma_3(t) - \sqrt{c_{13}[\mathrm{KIV}]}\,\Gamma_{13}(t)}{\sqrt{1+C_3[\mathrm{Val}]}} \quad (82)$$

$$v_4 = c_4[\mathrm{KIV}][\mathrm{Glut}] - c_{14}[\mathrm{Val}][\alpha\mathrm{KG}] + \sqrt{c_4[\mathrm{KIV}][\mathrm{Glut}]}\,\Gamma_4(t) - \sqrt{c_{14}[\mathrm{Val}][\alpha\mathrm{KG}]}\,\Gamma_{14}(t) \quad (83)$$

$$v_5 = c_5[\mathrm{KIV}][\mathrm{Ala}] - c_{15}[\mathrm{Val}][\mathrm{Pyr}] + \sqrt{c_5[\mathrm{KIV}][\mathrm{Ala}]}\,\Gamma_5(t) - c_{15}[\mathrm{Val}][\mathrm{Pyr}]\,\Gamma_{15}(t) \quad (84)$$

$$v_6 = \frac{c_6[\mathrm{Val}]}{1+C_4[\mathrm{Leu}]} + \sqrt{\frac{c_6[\mathrm{Val}]}{1+C_4[\mathrm{Leu}]}} \quad (85)$$

$$v_7 = \frac{c_7[\mathrm{KIV}][\mathrm{AcCoA}] - c_{17}[\mathrm{IPM}][\mathrm{CoA}]}{1+C_5[\mathrm{Leu}]} + \frac{\sqrt{c_7[\mathrm{KIV}][\mathrm{AcCoA}]} - \sqrt{c_{17}[\mathrm{IPM}][\mathrm{CoA}]}}{\sqrt{1+C_5[\mathrm{Leu}]}} \quad (86)$$

$$v_8 = c_8[\mathrm{IPM}][\mathrm{NAD^+}] - c_{18}[\mathrm{KIC}][\mathrm{NADH_2}] + \sqrt{c_8[\mathrm{IPM}][\mathrm{NAD^+}]} - \sqrt{c_{18}[\mathrm{KIC}][\mathrm{NADH_2}]} \quad (87)$$

$$v_9 = c_9[\mathrm{KIC}][\mathrm{Glut}] - c_{19}[\mathrm{Leu}][\alpha\mathrm{KG}] + \sqrt{c_9[\mathrm{KIC}][\mathrm{Glut}]} - \sqrt{c_{19}[\mathrm{Leu}][\alpha\mathrm{KG}]} \quad (88)$$

$$v_{10} = \frac{c_{10}[\mathrm{Leu}]}{1+C_6[\mathrm{Val}]} + \sqrt{\frac{c_{10}[\mathrm{Leu}]}{1+C_6[\mathrm{Val}]}}. \quad (89)$$

2.6 Optimization of the Model Parameters

This section briefly summarizes additional results of the parameter optimization that cannot be mentioned in the main article corresponding to this document.

The quality of a parameter set can be evaluated by taking the distance between simulated concentrations and a time series of measurements for each reacting species. Due to the high orders of magnitude for the concentrations of metabolites, the Relative Squared Error (RSE), which normalizes each distance by the measurement and is hence dimensionless, is used in this study:

$$f_{\mathrm{RSE}}(\hat{\mathbf{x}}, \mathbf{X}) = \sum_{i=1}^{\dim(\hat{\mathbf{x}})} \sum_{t=1}^{T} \left(\frac{\hat{x}_i(\tau_t) - x_{ti}}{x_{ti}} \right)^2. \quad (90)$$

This distance is often called the “fitness” of a possible solution. Table 2 gives an idea of how the relative distance of the best solutions for the deterministic models is computed. Each value

in this table is the inner sum of the RSE, i. e., the sum over all 47 time points for the respective metabolite. For each metabolite to be simulated, an independent spline is also computed using the same settings as described in Section 2.1. Table 2 also lists the relative distance between these splines and the measurements. The simulation results of the best solutions are shown in Figure 4 for the reversible models and Figure 5 for the irreversible models.

Metabolite	Spline	GMAKr	GMAKi	GMMr	GMMi	CKMMr	CKMMi
AcLac	1,437	1,861	1,417	1,849	2,055	1,339	1,453
DHIV	11,399	6,484	7,583	6,470	7,899	6,781	5,798
IPM	0,905	2,295	2,190	2,336	2,033	1,957	1,831
KIC	2,105	2,868	3,370	2,826	2,960	2,930	3,651
KIV	1,099	2,260	4,060	2,272	4,115	1,828	3,615
Leu	2,044	2,231	4,376	2,087	4,021	3,230	3,814
Val	0,680	2,327	1,591	2,441	1,394	2,035	1,347
$\sum$	19,670	20,326	24,587	20,280	24,477	20,100	21,511

Table 2: Computation of the fitness value for each deterministic model

To obtain these solutions we apply a random search (Monte Carlo Optimization, MCO) to calibrate the parameters for each model. As the results of this procedure cannot nearly approach the quality of independently fitted splines, we apply the nature-inspired heuristic optimization procedures Hill Climber (HC), Simulated Annealing (SA), real-valued and binary Genetic Algorithm (GA), standard and covariance matrix adaptation Evolution Strategy (ES) with and without elitism (plus strategy), Differential Evolution (DE), particle swarm optimization (PSO) and Tribes to all deterministic models with standard settings. Subsequently, the settings of the most promising procedures are systematically benchmarked to further improve the fit of the models to the measurements. The Langevin model is optimized using the most successful methods from the fine-tuning step. For details of the optimization procedures, [see Additional file 1]. All optimization procedures, models and the data used in this study are freely available in the optimization framework EvA2 [21, 22].

Figure 6 shows the progress of the five most successful optimization procedures with their standard settings in 100,000 fitness evaluations. Each procedure is started in twenty multi-runs, of which the best is depicted here. The quality of the initial fitness rises with the complexity of the model. All plots are limited to a fitness of 120 for the sake of a better visualization. In most cases, the settings-free Tribes algorithm needs more fitness evaluations than the other methods to find a good solution. And in most cases again, the algorithms do not show significant improvement after 60,000 evaluations.

From the set of parameter vectors obtained during the optimization process, we select all parameter vectors with a fitness less than 25, focusing on the three deterministic reversible models. Figures 7 through 9 give histograms that depict the distribution of the parameters. All models have in common that most parameter values are rather small.

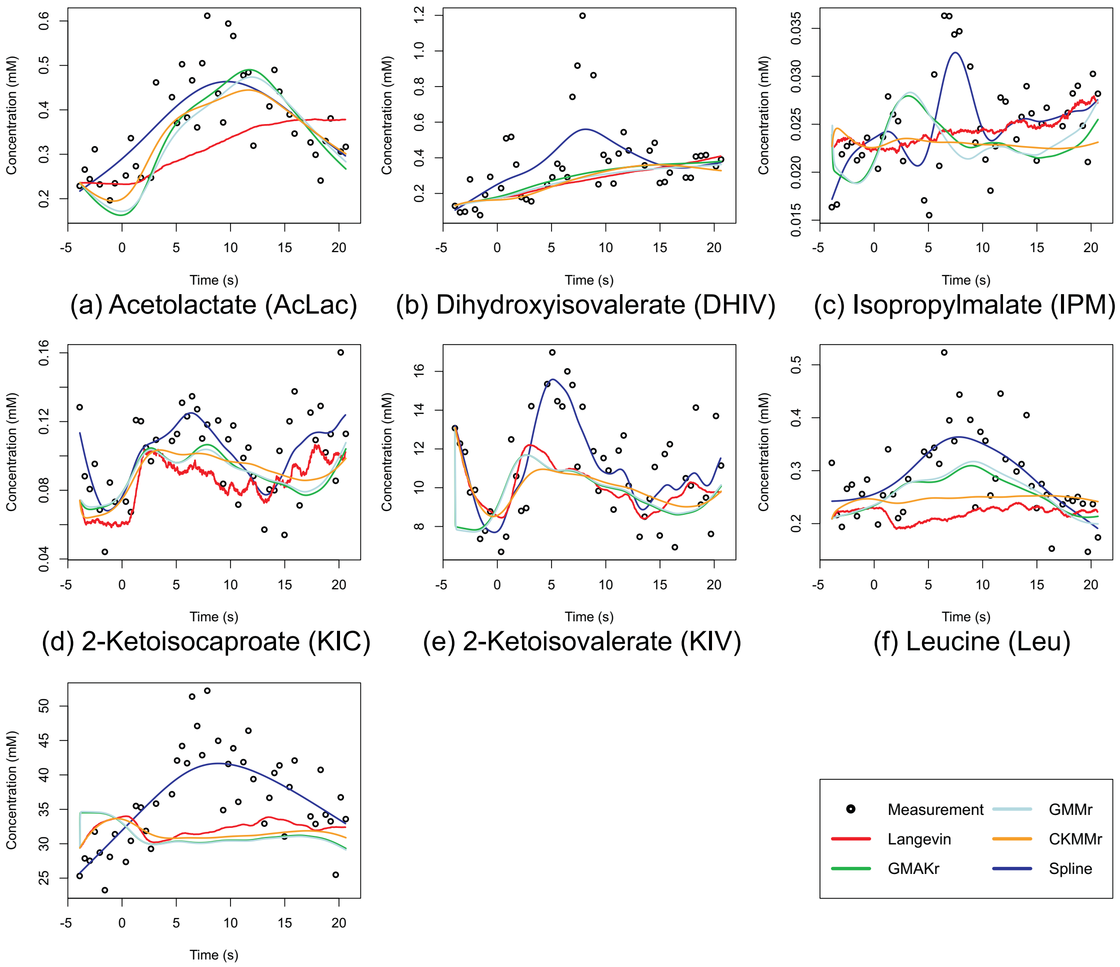

(a) Acetolactate (AcLac) (b) Dihydroxyisovalerate (DHIV) (c) Isopropylmalate (IPM)

(d) 2-Ketoisocaproate (KIC) (e) 2-Ketoisovalerate (KIV) (f) Leucine (Leu)

(g) Valine (Val)

Figure 4: The best fit of all reversible deterministic and the Langevin models

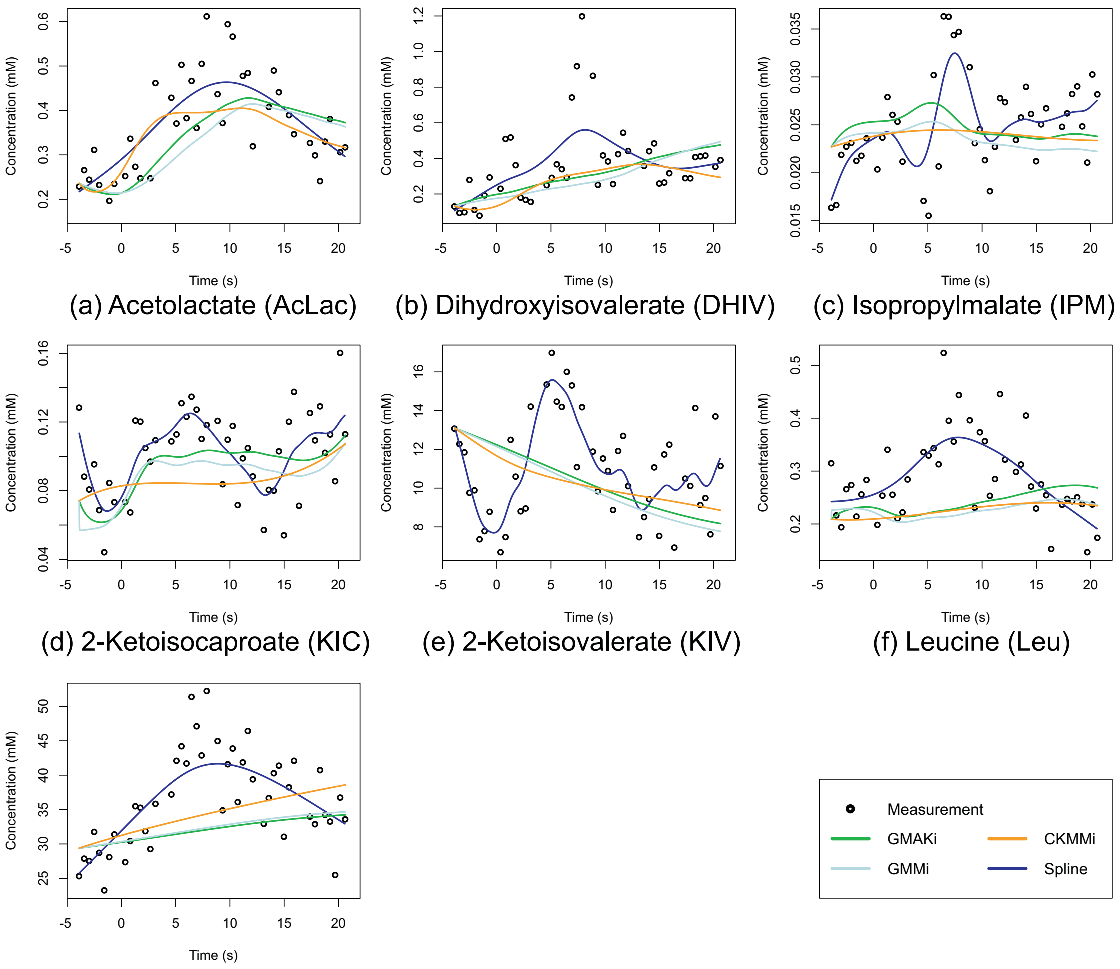

(a) Acetolactate (AcLac)

(b) Dihydroxyisovalerate (DHIV)

(c) Isopropylmalate (IPM)

(d) 2-Ketoisocaproate (KIC)

(e) 2-Ketoisovalerate (KIV)

(f) Leucine (Leu)

(g) Valine (Val)

Figure 5: The best fit of all irreversible deterministic models

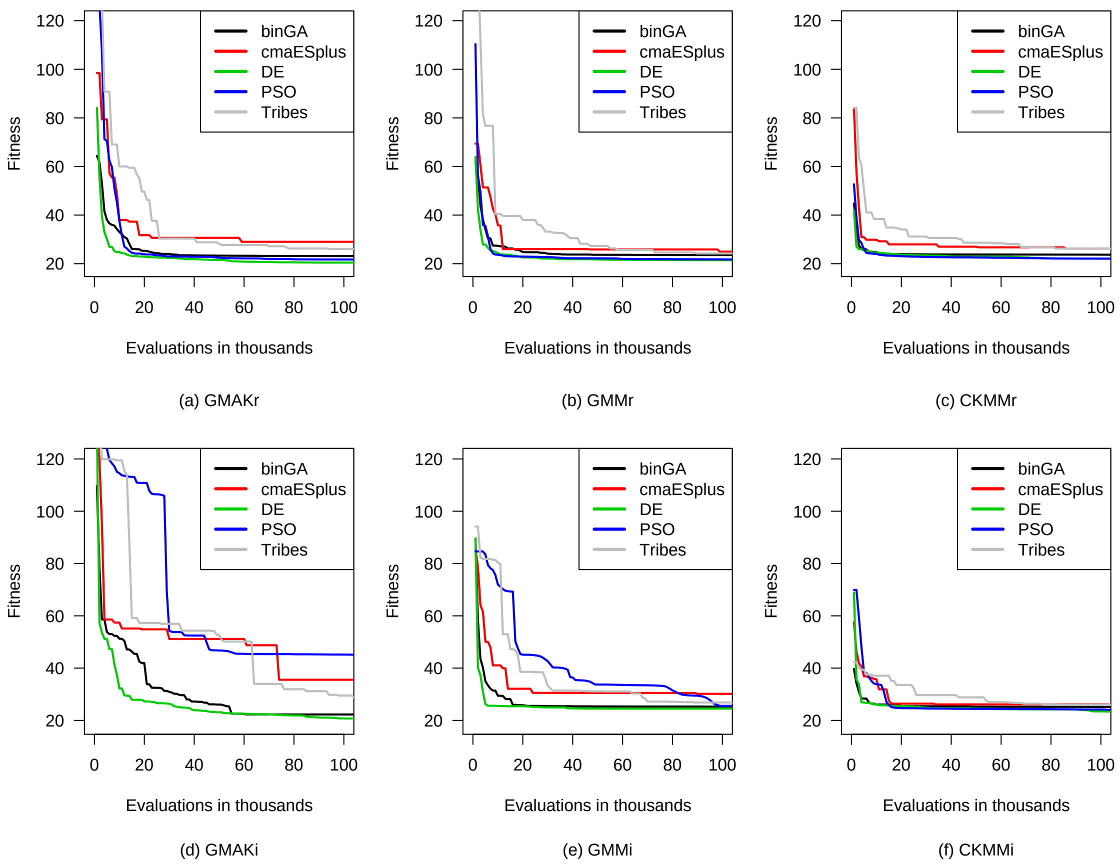

Figure 6: Progress of the optimization algorithms

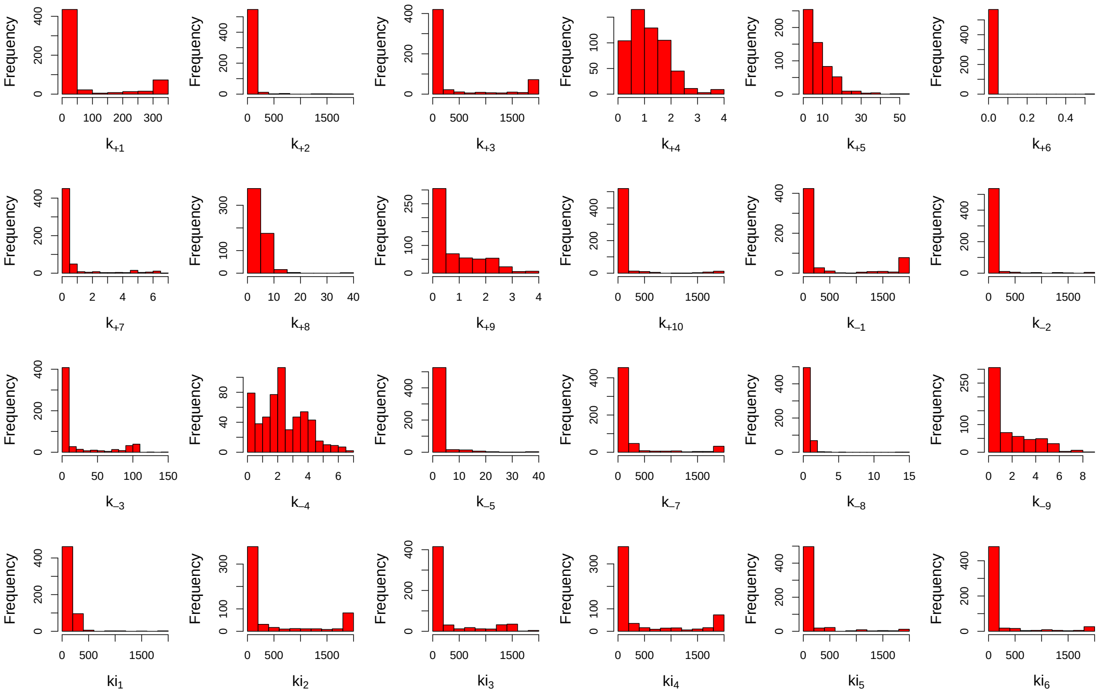

Figure 7: Histograms of the parameter distribution for the GMAKr model

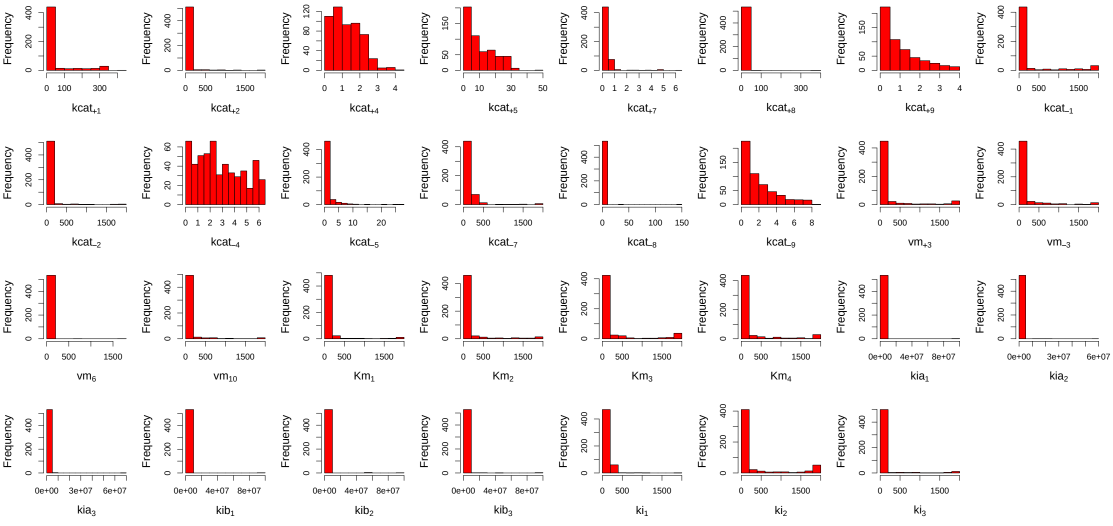

Figure 8: Histograms of the parameter distribution for the GMMr model

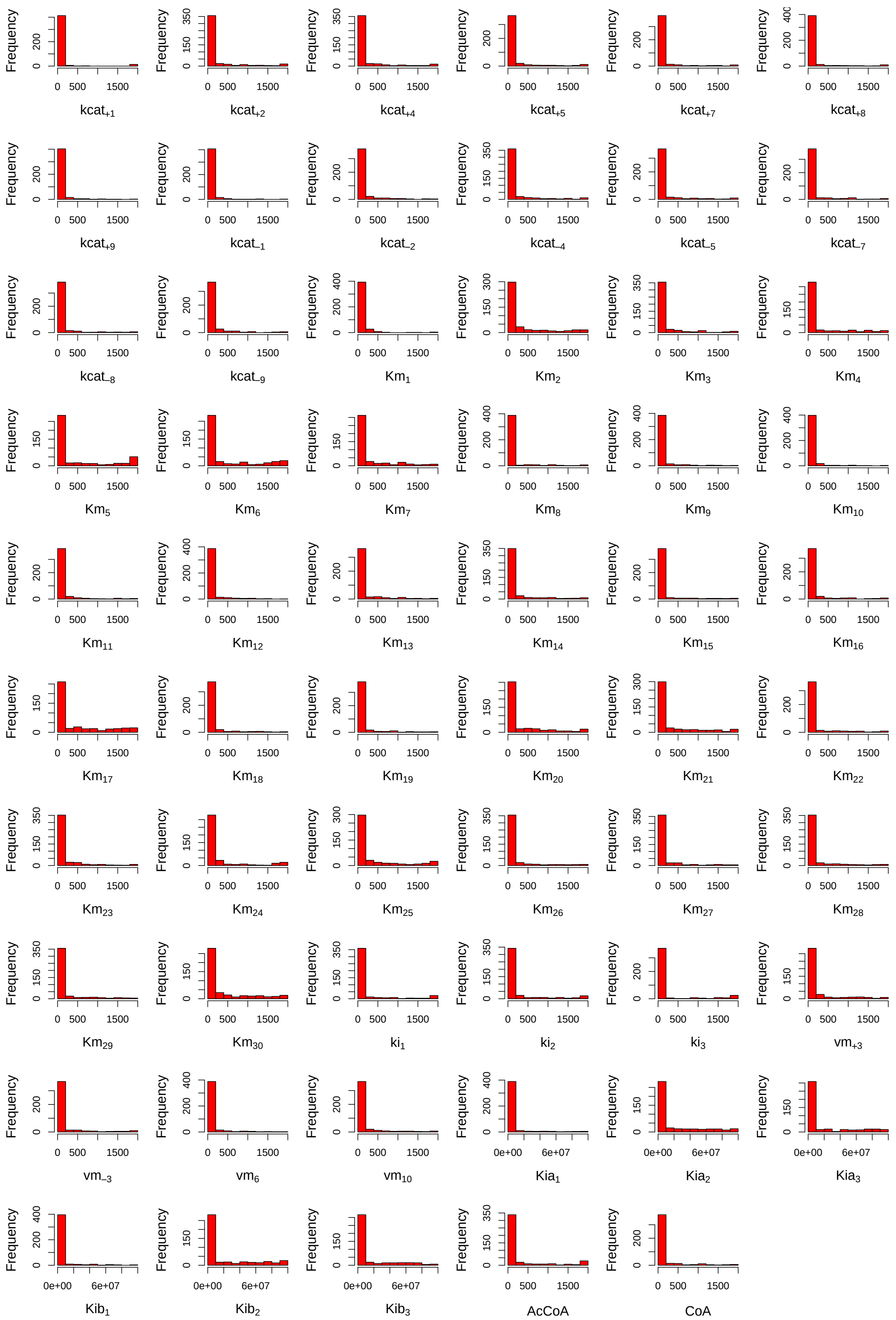

Figure 9: Histograms of the parameter distribution for the CKMMr model

References

[1] Ogata H, Goto S, Sato K, Fujibuchi W, Bono H, Kanehisa M: **KEGG: Kyoto Encyclopedia of Genes and Genomes**. *Nucleic Acids Research* 2000, **27**:29–34.

[2] Kanehisa M, Goto S, Hattori M, Aoki-Kinoshita KF, Itoh M, Kawashima S, Katayama T, Araki M, Hirakawa M: **From genomics to chemical genomics: new developments in KEGG**. *Nucl. Acids Res.* 2006, **34**:D354–357, [http://nar.oxfordjournals.org/cgi/content/abstract/34/suppl_1/D354].

[3] Caspi R, Foerster H, Fulcher CA, Hopkinson R, Ingraham J, Kaipa P, Krummenacker M, Paley S, Pick J, Rhee SY, Tissier C, Zhang P, Karp PD: **MetaCyc: a multiorganism database of metabolic pathways and enzymes.** *Nucleic Acids Research* 2006, **34**(Database issue):D511–D516, [http://dx.doi.org/10.1093/nar/gkj128].

[4] Bulik S, Grimbs S, Selbig J, Holzhütter HG: **Combining mechanistic and simplified enzymatic rate equations: A promising approach for speeding up the kinetic modeling of complex metabolic networks** 2008.

[5] Albert R: **Network Inference, Analysis, and Modeling in Systems Biology**. *Plant Cell* 2007, **19**(11):3327–3338, [http://www.plantcell.org/cgi/reprint/19/11/3327.pdf].

[6] Gillespie DT: **The chemical Langevin equation**. *Journal of Chemical Physics* 2000, **113**:297–306, [http://adsabs.harvard.edu/cgi-bin/nph-bib_query?bibcode=2000JChPh.113..297G&db_key=PHY.

[7] Gillespie DT: **A General Method for Numerically Simulating the Stochastic Time Evolution of Coupled Chemical Reactions**. *Journal of Computational Physics* 1976, **22**(4):403–434, [http://www.sciencedirect.com/science/article/B6WHY-4DD1NC9-CP/2/43ade5f11fb949602b3a2abdbbb29f0e].

[8] Savageau MA: **Biochemical systems analysis. II. The steady-state solutions for an n-pool system using a power-law approximation.** *J Theor Biol* 1969, **25**(3):370–379.

[9] Savageau MA: **Biochemical systems analysis. 3. Dynamic solutions using a power-law approximation**. *J Theor Biol* 1970, **26**(2):215–226.

[10] Heinrich R, Schuster S: *The Regulation of Cellular Systems*. 115 Fifth Avenue New York, NY 10003: Chapman and Hall 1996.

[11] Visser D, Heijnen J: **The Mathematics of Metabolic Control Analysis Revisited**. *Metabolic Engineering* 2002, **4**:114–123, [http://www.sciencedirect.com/science/article/B6WN3-45V802C-3/2/d624a20d0e70ca2a1058359d7fd00cb0].

[12] Hatzimanikatis V, Floudas CA, Bailey JE: **Analysis and design of metabolic reaction networks via mixed-integer linear optimization**. *AIChE* 1996, **42**(5):1277–1292.

[13] Liebermeister W, Klipp E: **Bringing metabolic networks to life: convenience rate law and thermodynamic constraints.** *Theor Biol Med Model* 2006, **3**:41, [`http://dx.doi.org/10.1186/1742-4682-3-41`].

[14] Schauer M, Heinrich R: **Quasi-steady-state approximation in the mathematical modeling of biochemical reaction networks**. *Mathematical Bioscience* 1983, **65**:155–171.

[15] Cornish-Bowden A: *Fundamentals of Enzyme Kinetics*. 59 Portland Place, London: Portland Press Ltd., 3rd edition 2004.

[16] Gillespie D: **A rigorous derivation of the chemical master equation**. *Physica A* 1992, **188**:404–425.

[17] Gillespie DT: **Stochastic simulation of chemical kinetics**. *Annu Rev Phys Chem* 2007, **58**:35–55, [`http://dx.doi.org/10.1146/annurev.physchem.58.032806.104637`].

[18] Kloeden PE, Platen E: *Numerical Solution of Stochastic Differential Equations*. Applications of Mathematics, Berlin: Springer-Verlag 1992.

[19] Bentele M: **Stochastic Simulation and System Identification of large Signal Transduction Networks in Cells**. *PhD thesis*, Combined Faculties for the Natural Sciences and for Mathematics of the Ruperto-Carola University Heidelberg, Germany 2004.

[20] Magnus JB, Hollwedel D, Oldiges M, Takors R: **Monitoring and Modeling of the Reaction Dynamics in the Valine/Leucine Synthesis Pathway in *Corynebacterium glutamicum***. *Biotechnology Progress* 2006, **22**(4):1071–1083, [`http://dx.doi.org/10.1021/bp060072f`].

[21] Kronfeld M: *EvA2 Short Documentation*. University of Tübingen, Dept. of Computer Architecture, Sand 1, 72076 Tübingen, 2008, [`http://www.ra.cs.uni-tuebingen.de/software/EvA2`].

[22] Streichert F, Ulmer H: **JavaEvA—A Java Framework for Evolutionary Algorithms**. Technical Report WSI-2005-06, Center for Bioinformatics Tübingen, University of Tübingen 2005, [`http://w210.ub.uni-tuebingen.de/dbt/volltexte/2005/1702/`].
